# Single-atom photocatalyst for click reaction

**DOI:** 10.1038/s41467-026-74278-4

**Published:** 2026-06-10

**Authors:** Chang Cheng, Bicheng Zhu, Yue Lin, Zhifeng Jiang, Guijie Liang, Chuanjia Jiang, Hermenegildo García, Jiaguo Yu

**Affiliations:** 1 Laboratory of Solar Fuel, Faculty of Materials Science and Chemistry, China University of Geosciences, 68 Jincheng Street, Wuhan, 430078 P. R. China; 2 Hefei National Research Center for Physical Sciences at the Microscale, University of Science and Technology of China, 96 Jinzhai Road, Hefei, 230026 P. R. China; 3 Institute for Energy Research, School of Chemistry and Chemical Engineering, Jiangsu University, Zhenjiang, 212013 P. R. China; 4 Hubei Key Laboratory of Low Dimensional Optoelectronic Material and Devices, Hubei University of Arts and Science, Xiangyang, 441053 P. R. China; 5 College of Environmental Science and Engineering, Nankai University, 38 Tongyan Road, Tianjin, 300350 P. R. China; 6 Instituto Universitario de Tecnología Química, CSIC-UPV, Universitat Politècnica de València, Valencia, Spain

**Keywords:** Photocatalysis, Photocatalysis, Synthetic chemistry methodology

## Abstract

Copper(I)-catalyzed azide-alkyne cycloaddition is the most prevalent click chemistry reaction, which is widely exploited for the synthesis of diverse organic molecules. Typically, the Cu(I) catalysts are generated in situ through the chemical reduction of Cu(II) ions. Unfortunately, such homogeneous systems are considered impractical for large-scale production, and the involvement of multiple equilibria among catalysts, reductants, and substrates complicates elucidation of the catalytic process. Inspired by photosynthesis, which involves transient redox cycle of magnesium porphyrin moiety in chlorophylls, a metal complex-based strategy is proposed for light-triggered click chemistry reactions, utilizing a single-atom Cu(II)-loaded covalent organic framework as a heterogeneous catalyst, wherein the organic ligand functions as a robust scaffold and photoinitiator. Upon light irradiation, photogenerated electrons within the ligand moiety undergo ligand-to-metal charge transfer, injecting into the 3 *d* orbital of Cu(II) and reducing it to Cu(I) as a short-lived transient species with submicrosecond lifetime, which acts as efficient active sites for the click chemistry reaction. This controllable and robust system shows promise in photochemical cycloaddition of benzylazide and phenylacetylene, a model click chemistry reaction, achieving 95% substrate conversion and >90% triazole yield. The findings are anticipated to be broadly applicable in areas such as sustainable photolithography and polymer chemistry.

## Abstract (web summary)

Copper(I)-catalyzed azide–alkyne cycloaddition is one of the most widely used click chemistry reactions, but conventional homogeneous systems suffer from poor scalability and mechanistic complexity. Inspired by photosynthetic redox cycling, the authors develop a single-atom Cu(II)-loaded covalent organic framework that enables light-triggered generation of transient Cu(I) active sites, achieving efficient heterogeneous cycloaddition with high conversion and yield.

## Introduction

Click chemistry has been widely exploited for the rapid and precise synthesis of diverse organic molecules^1,2^. Among various click chemistry reactions^3–5^, copper(I)-catalyzed alkyne-azide cycloaddition (CuAAC) stands out for its high efficiency and versatility, making it the most powerful technique for applications such as bioconjugation and polymer modification^6,7^. Due to its instability upon exposure to air, Cu(I) catalysts are typically generated in situ from Cu(II) using chemical reductants. To prevent the undesirable re-oxidation of Cu(I) to Cu(II), these reductants are typically used in large excess, which adds cost and is environmentally unfriendly. Inspired by photosynthesis, which involves a transient redox cycle of magnesium porphyrin moiety in chlorophylls^8^, photochemical Cu(I) generation offers a greener alternative, and, more importantly, enables spatial and temporal control of reaction onset, which is vital for applications such as photolithography and post-polymerization modification^9,10^.

In photochemical CuAAC, Cu(I) is generated predominantly via indirect photoreduction using sacrificial electron donors, which relies on electron transfer to Cu(II) via photoexcited intermediates. However, this approach entails complex interfacial charge and mass transfer processes between multiple components, complicating rigorous mechanistic elucidation of the photocatalytic cycle. Furthermore, the low reduction potentials of free Cu ions [+0.159 V from Cu(II) to Cu(I) and +0.520 V from Cu(I) to Cu(0)] strictly constrain the selection of photoinitiators and often lead to irreversible formation of inactive Cu(0) aggregates^11,12^. To circumvent these limitations, the photogeneration of transient Cu(I) within Cu(II) complexes—particularly in polymer-based single-atom systems—emerges as a promising strategy. This approach enables spontaneous return to Cu(II) after having completed a catalytic cycle. Besides, such heterogeneous photocatalysts can be easily separated from the reaction system and recycled without special purification, which is conducive to their industrial application. More importantly, in photoinduced Cu(I) generation by irradiation of Cu(II) complexes, intramolecular ligand-to-metal charge transfer (LMCT) is a more efficient and controllable process than charge transfer between two components [i.e., photoinitiators and Cu(II) salts] with random positions and instantaneously changing distances^13–16^. Thus, direct photoreduction of Cu(II) complexes to generate transient Cu(I) species is worth exploring as a controllable and scalable platform for photochemical CuAAC.

To this end, we synthesized a single-atom Cu(II)-loaded covalent organic framework (COF) as a heterogeneous catalyst for light-triggered CuAAC, wherein the COF ligand acts as a robust scaffold and an efficient photoinitiator^17^. Upon irradiation, the ligand moiety [1,3,5-tris(4-aminophenyl)benzene-*alt*-bipyridine, TPB-BPy] absorbs photons, while electrons are excited from the π bonding orbitals to π antibonding orbitals (π*). The π* electrons can easily transfer to the 3 *d* orbitals of Cu(II), reducing it to Cu(I). In its short lifetime, transient Cu(I) performs as active sites for CuAAC. In this way, TPB-BPy-Cu(II) acts as a heterogeneous CuAAC catalyst without the need for any sacrificial electron donor. The physicochemical properties of TPB-BPy-Cu and metal-free TPB-BPy (a reference sample) were thoroughly characterized, with the fine structural features of the single-atom Cu site precisely elucidated by using X-ray absorption spectroscopy (XAS). More importantly, the LMCT process was elucidated through steady-state spectral studies and theoretical calculations, while the charge-transfer dynamics were explored via femtosecond transient absorption (fs-TA) spectroscopy. This investigation aims to provide a new platform for efficient click chemistry reactions and advance the mechanistic understanding of single-atom photocatalysts for organic transformations.

## Results

### Characterization of TPB-BPy-Cu with single Cu(II) atoms

**Fig. 1:**
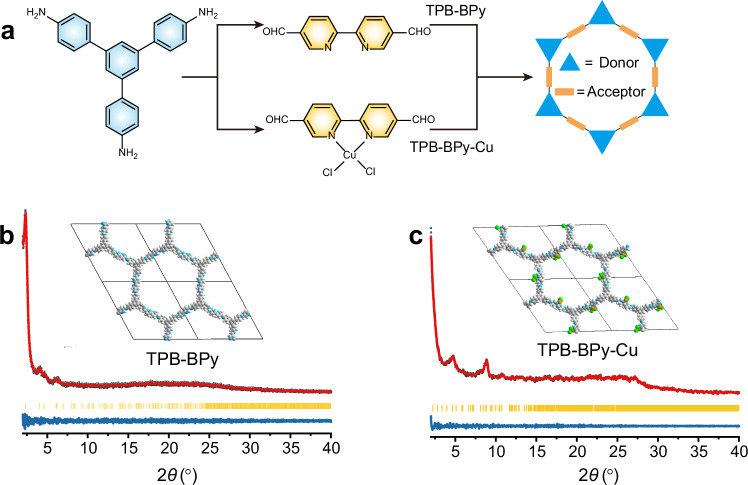
Synthesis and structures. **a** Polycondensation routes for synthesizing TPB-BPy and TPB-BPy-Cu. **b, c** XRD patterns of TPB-BPy and TPB-BPy-Cu, including the experimental (black), Pawley refined (red), Bragg positions (yellow), and the differences between the Pawley refined and experimental patterns (blue). Insets in parts b and c show structures of the TPB-BPy and TPB-BPy-Cu.

To obtain the COF with monodisperse Cu atoms, Cu(II) was first coordinated to 2,2’-bipyridine-5,5’-dicarbaldehyde (BPy), with the Cu(II)-coordinated structure confirmed by mass spectroscopy (Supplementary Fig. 1)^18^. The BPy-Cu complex was then coupled with 1,3,5-tris(4-aminophenyl)benzene (TPB) via Schiff-base polycondensation, yielding TPB-BPy-Cu (Fig. 1a). A metal-free TPB-BPy COF was synthesized for comparison. The successful polymerization was assessed by Fourier transform infrared spectroscopy (FTIR, Supplementary Fig. 2a)^19^. Both COFs feature AA stacking two-dimensional (2D) structures, as revealed by their X-ray diffraction (XRD) patterns (Fig. 1b, c)^20,21^. TPB-BPy exhibits a triclinic P1 space group with lattice parameters *a* = 44.10 Å, *b* = 44.95 Å, and *c* = 3.60 Å and an interlayer spacing of 4.55 Å (Supplementary Fig. 2b), while TPB-BPy-Cu shows similar triclinic symmetry with altered lattice parameters (*a* = 46.00 Å, *b* = 44.26 Å, and *c* = 3.93 Å) and a reduced π-π stacking distance of 3.41 Å^22^. X-ray photoelectron spectroscopy (XPS, Supplementary Figs. 3 and 4) confirms the interaction between Cu and the ligand.

**Fig. 2:**
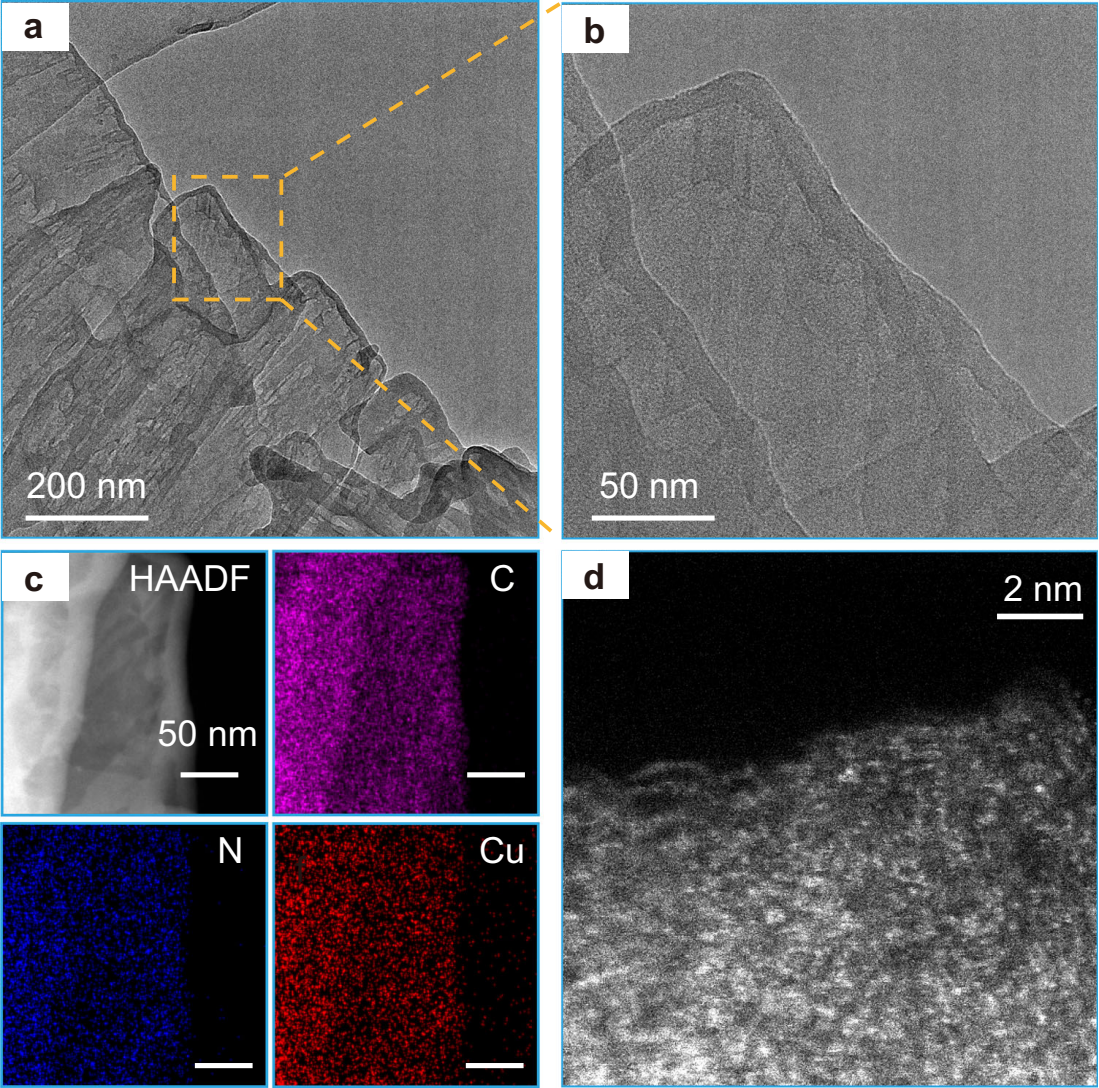
Morphology of TPB-BPy-Cu. **a** The transmission electron microscopy (TEM) and (**b**) high-resolution TEM images of TPB-BPy-Cu. **c** HAADF-STEM image and corresponding elemental mapping of TPB-BPy-Cu. **d** The atomic HAADF-STEM image of the TPB-BPy-Cu sample.

**Fig. 3:**
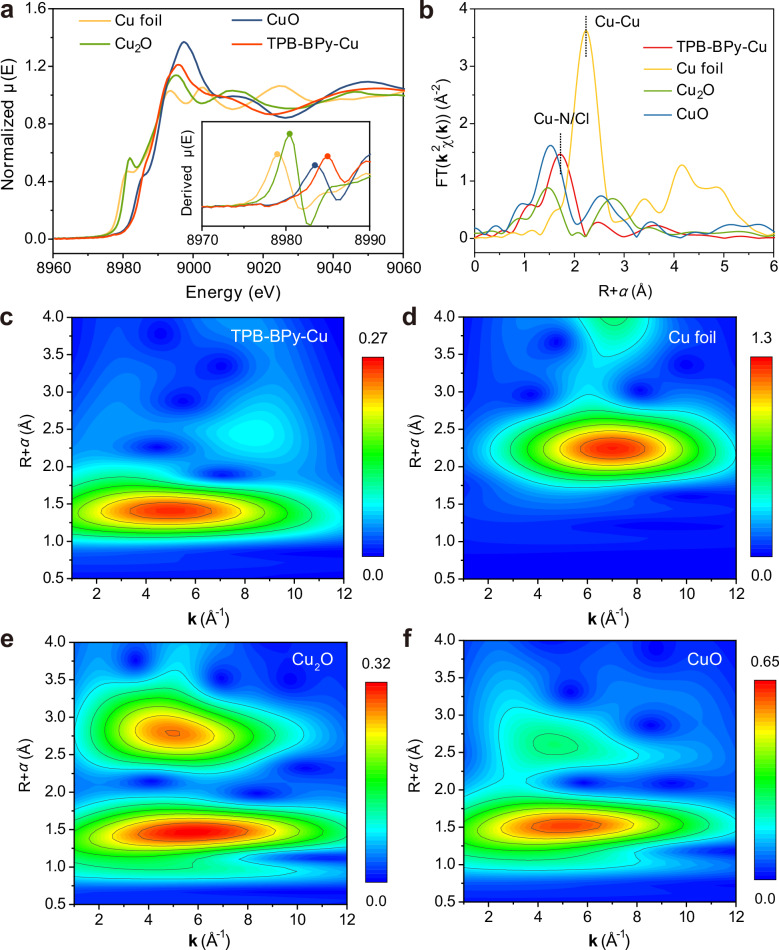
Coordiantion structure of samples. **a** Cu K-edge XANES spectra of TPB-BPy-Cu and the reference samples Cu foil, Cu_2_O, and CuO (The inset shows the derived curves of XANES spectra). **b** Cu K-edge EXAFS spectrum for TPB-BPy-Cu and the reference samples in **k**^2^-weighted *R*-space. **c–f** Wavelet transform EXAFS contour plots of TPB-BPy-Cu and the reference samples.

The presence of monodisperse Cu was confirmed using high-angle annular dark-field scanning transmission electron microscopy (HAADF-STEM)^23^. As shown in Fig. 2a, b, no Cu nanoparticle is observed in TPB-BPy-Cu, but abundant Cu element is distributed over its surface, indicating that Cu atoms are not aggregated (Fig. 2c). Upon observation of the atomic HAADF-STEM (Fig. 2d), multiple bright dots with a size of ~0.1 nm are dispersed on the plate-like substrate, and each of them corresponds to an individual Cu atom. The X-ray absorption near edge structure (XANES) spectra (Fig. 3a) show that the Cu K-edge position for TPB-BPy-Cu is 8983 eV, which is close to the pre-edge of CuO (8982 eV) and significantly higher than those of Cu_2_O (8980 eV) and Cu foil (8977 eV)^24^. Therefore, the pristine TPB-BPy-Cu is identified as a Cu(II) complex^3^, in line with the calculated result (Supplementary Table 1). The coordination environment of Cu(II) was investigated using extended X-ray absorption fine structure (EXAFS) analysis, with the **k**^2^-weighted EXAFS **k**-space data shown in Supplementary Fig. 5. Corresponding *R*-space data (Fig. 3b) reveal a main peak at 1.7 Å (without phase-correction), attributed to Cu-N and Cu-Cl bonds. The main peaks of CuO and Cu_2_O are also located in the first shell (1-2 Å), whereas the main peak of Cu foil is located at 2.3 Å, which reflects the Cu-Cu bonds. Wavelet transform analysis of TPB-BPy-Cu (Fig. 3c) shows an intensity maximum at 5.7 Å^−1^ in the first shell, which is attributed to the scattering from Cu atoms directly bonded with N and Cl atoms^25^. By contrast, the wavelet transform EXAFS contour plots of Cu foil, CuO, and Cu_2_O (Fig. 3d–f) exhibit distinct signals in the second shell. EXAFS fitting (Supplementary Fig. 6) indicates a coordination number of 4 (2 Cu-N, 2 Cu-Cl), with Cu-N and Cu-Cl bond lengths of 1.98 and 2.24 Å, respectively (Supplementary Table 2). Collectively, these results confirm the successful synthesis of TPB-BPy-Cu with single Cu(II) atoms coordinated with N and Cl atoms.

### Photoinduced reduction of Cu(II) to Cu(I) via LMCT

**Fig. 4:**
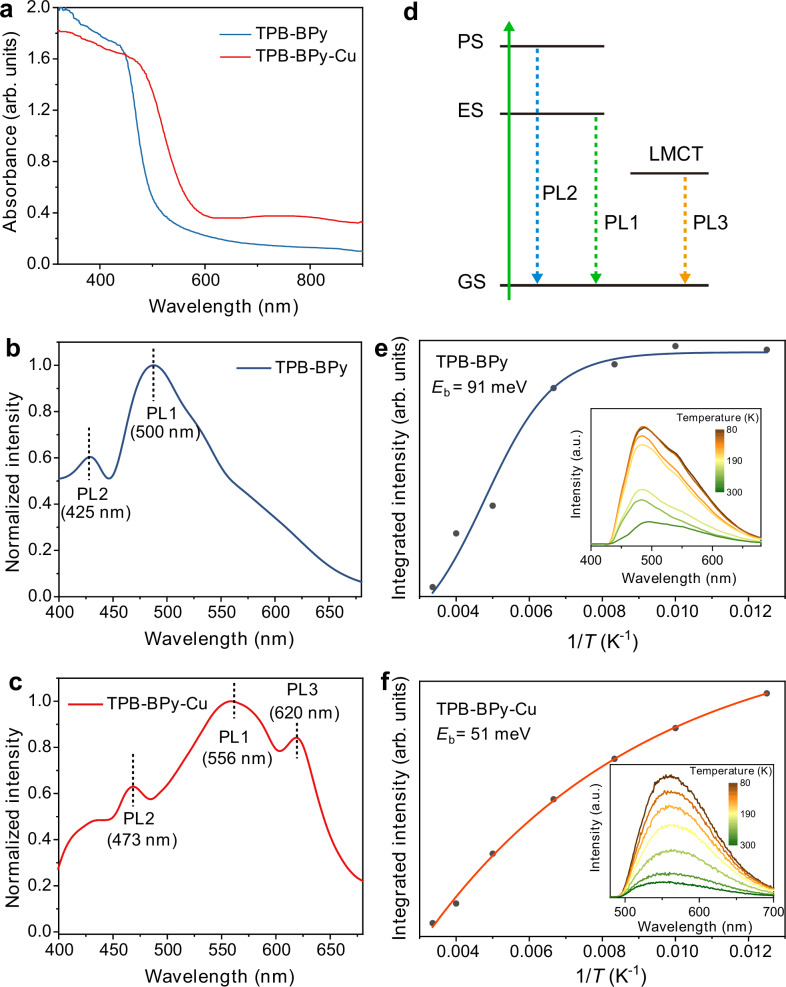
Spectral studies on the photophysical processes. **a** Absorption spectra of TPB-BPy and TPB-BPy-Cu. Steady-state PL spectra of (**b**) TPB-BPy and (**c**) TPB-BPy-Cu, where PL1, PL2, and PL3 denote the observed PL peaks. **d** Schematic illustration of radiative recombination processes within the COFs. Integrated PL emission intensity of (**e**) TPB-BPy and (**f**) TPB-BPy-Cu at different temperatures. Inset in parts e and f: temperature-dependent PL spectra of the samples.

The band structures and photophysical properties of the materials were investigated using steady-state absorption and photoluminescence (PL) spectroscopies. TPB-BPy shows strong visible light absorption (π → π* transition) with an optical gap of 2.58 eV (Fig. 4a and Supplementary Fig. 7)^26^, and its HOMO and LUMO levels are −6.31 and −3.73 eV, respectively (Supplementary Fig. 8). The significant dipole moment of TPB-BPy can trigger intramolecular charge transfer (ICT, Supplementary Fig. 9a, b) and polarized excitons^27–29^. As a result, TPB-BPy exhibits two emission bands at 500 nm (PL1, Fig. 4b) and 425 nm (PL2), attributed to the relaxations of the exciton state (ES) and polarization state (PS) to the ground state (GS), respectively^30^. Upon Cu coordination, the molecular electron push-pull effect is promoted, which bestows TPB-BPy-Cu a narrowed optical gap of 2.25 eV and red-shifted PL emission^31^. The PL spectrum of TPB-BPy-Cu exhibits three emission bands (Fig. 4c). The emission bands at 556 nm (PL1) and 473 nm (PL2) correspond to relaxation of ES and PS, while the new band at 620 nm (PL3) is attributed to deexcitation of LMCT state [*d*(Cu)→π, Fig. 4d]^32^.

The LMCT process is conducive to separation of excitons within TPB-BPy-Cu, as elucidated through temperature-dependent PL spectroscopy (Fig. 4e, f)^33,34^. As the temperature decreases, the PL1 peak of TPB-BPy and TPB-BPy-Cu increases monotonically. Their exciton binding energies (*E*_b_) can be derived by fitting the data to the Arrhenius equation. The *E*_b_ of TPB-BPy is 91 meV, which is comparable to the reported values for polymeric photocatalysts^33,35^. The coordination of Cu reduces the *E*_b_ to 51 meV, suggesting that the excitons within TPB-BPy-Cu are more easily separable.

**Fig. 5:**
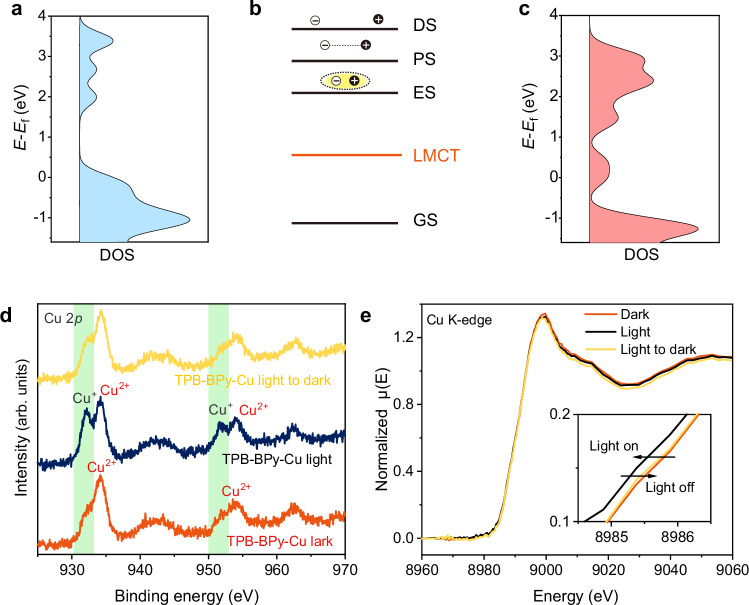
Insight into the LMCT process. **a** DOS of the TPB-BPy fragment. **b** Extracted energy levels from the DOS. **c** DOS of the TPB-BPy-Cu fragment. **d** In situ irradiated XPS spectra of the Cu 2*p* core level in TPB-BPy-Cu and (**e**) in situ irradiated Cu K-edge XANES spectra of TPB-BPy-Cu under dark-light-dark conditions.

The LMCT process was further revealed using density functional theory (DFT) calculations. As shown in Fig. 5a, TPB-BPy exhibits four high-density of states (DOS) near the Fermi level (*E*_f_)^36,37^, corresponding to the ground, exciton, polarization, and dissociated states (DS, Fig. 5b), respectively. Upon Cu coordination (Fig. 5c), the four bands still exist, while a new band appears, stemming from the interaction between the ligand and Cu atoms. In situ irradiated XPS analysis provides compelling evidence for such interaction. The Cu 2*p* spectrum exhibits two major peaks at 934.1 and 953.8 eV under dark conditions (Fig. 5d), corresponding to Cu(II)^38^. Interestingly, a new set of Cu(I) signals located at 932.2 and 951.6 eV emerged upon illumination (*λ* = 365 nm) and disappeared when the light was turned off, demonstrating that the Cu(II) in TPB-BPy-Cu can be photoreduced to Cu(I) via a reversible LMCT process. Similar results can be obtained through an in situ irradiated Cu K-edge XANES test. As shown in Fig. 5e, the Cu K-edge was negatively shifted upon light (*λ* = 365 nm) illumination and recovered to the initial state upon turning off the light, further confirming the presence of LMCT^39^.

**Fig. 6:**
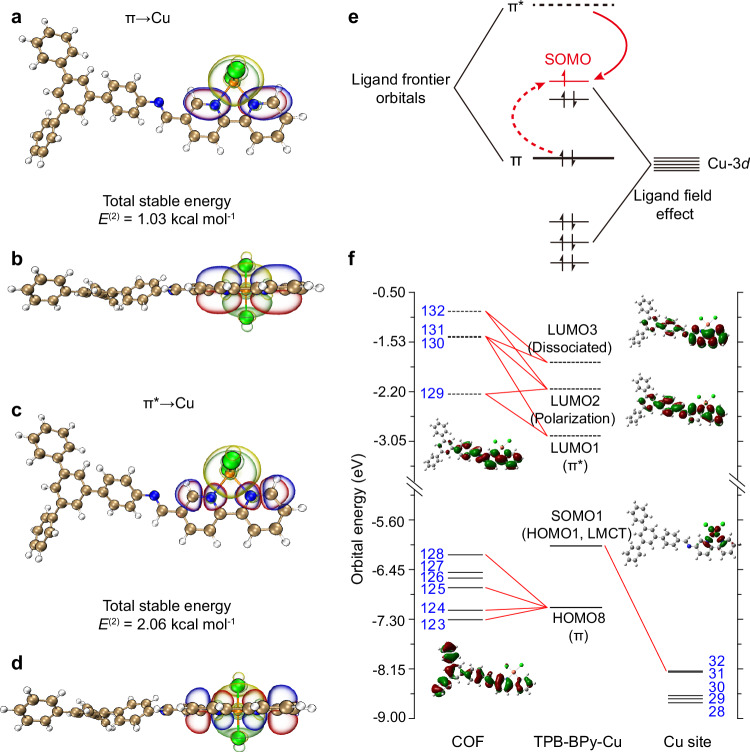
Orbital understanding of the LMCT process. **a, b** NBO analysis of the interaction between π and Cu-centered orbitals (isovalue = ±0.05) from (**a**) top view and (**b**) front view. **c, d** NBO analysis of the interaction between π* and Cu-centered orbitals (isovalue = ±0.05) from (**c**) top view and (**d**) front view. **e** The schematic electronic structure diagram with metal site and ligand orbitals. **f** Charge decomposition analysis on the TPB-*alt*-BPy-Cu fragment (isovalue = ±0.03).

Such ligand-to-metal electron injection can potentially proceed via two distinct pathways: direct photoexcitation from the ligand π orbital to the Cu atom (π → Cu), or a transfer from the ligand’s excited π* orbital to the Cu atom (π*→Cu). To elucidate the dominant mechanism, we employed natural bond orbital (NBO, Fig. 6a–d) analysis to quantify the wavefunction overlap and interaction intensity between the ligand π/π* orbitals and the Cu-centered orbitals. Quantified by second-order perturbation theory, the stabilization energy [*E*^(2)^] for the π → Cu interaction was found to be 1.03 kcal mol^−1^. In stark contrast, the stabilization energy for the π*→Cu interaction was significantly higher (2.06 kcal mol^−1^). This substantial difference in stabilization energy conclusively indicates that the π*→Cu transfer represents the predominant pathway for the LMCT (Fig. 6e). Deeper insight into electron transfer to Cu(II) via the LMCT process was gained by examining the atomic orbital contributions to the energy states through charge decomposition analysis^40,41^. As shown in Fig. 6f, the molecular orbitals of TPB-BPy-Cu consist of two fragments: the orbitals of the COF ligand and the Cu site. Among them, the HOMO8 of TPB-BPy-Cu fully consists of the HOMOs of the ligand; thus, the HOMO8 is the π bonding orbital and corresponds to the GS. Similarly, the LUMO1, LUMO2, and LUMO3 fully derive from the LUMOs of the ligand and serve as the excited states of TPB-BPy-Cu. Specifically, LUMO1, which has the lowest energy, is the π* orbital and corresponds to the ES, while LUMO2 and LUMO3 are attributed to the PS and DS, respectively. Several orbitals (HOMO1 to HOMO7) exist between LUMO1 and HOMO8, wherein HOMO1 is mainly contributed by the Cu orbital, and the others are hybrids of the ligand and Cu orbitals. According to the ligand field theory, the degenerate 3 *d* orbitals of Cu(II) can undergo redistribution and split into five electron orbitals, which corresponds to the 28th to 32nd orbitals of Cu(II). Given that the electron configuration of Cu(II) is 3 *d*^9^, the 28th to the 31st orbitals are fully occupied, whereas the 32nd orbital harbors an unpaired electron, known as the singly occupied molecular orbital (SOMO)^42^. Thus, the HOMO1 state is primarily attributed to the 32nd orbital of Cu, representing the highest SOMO (i.e., SOMO1) of TPB-BPy-Cu. In this scenario, SOMO1 is the only occupied orbital capable of accepting an additional electron, thus contributing to the LMCT state (Supplementary Fig. 9c). Upon the transition from LUMO1 to SOMO1, the electron can be injected into the split 3*d* orbital, thus reducing Cu(II) to Cu(I).

### Photophysical process dynamics

**Fig. 7:**
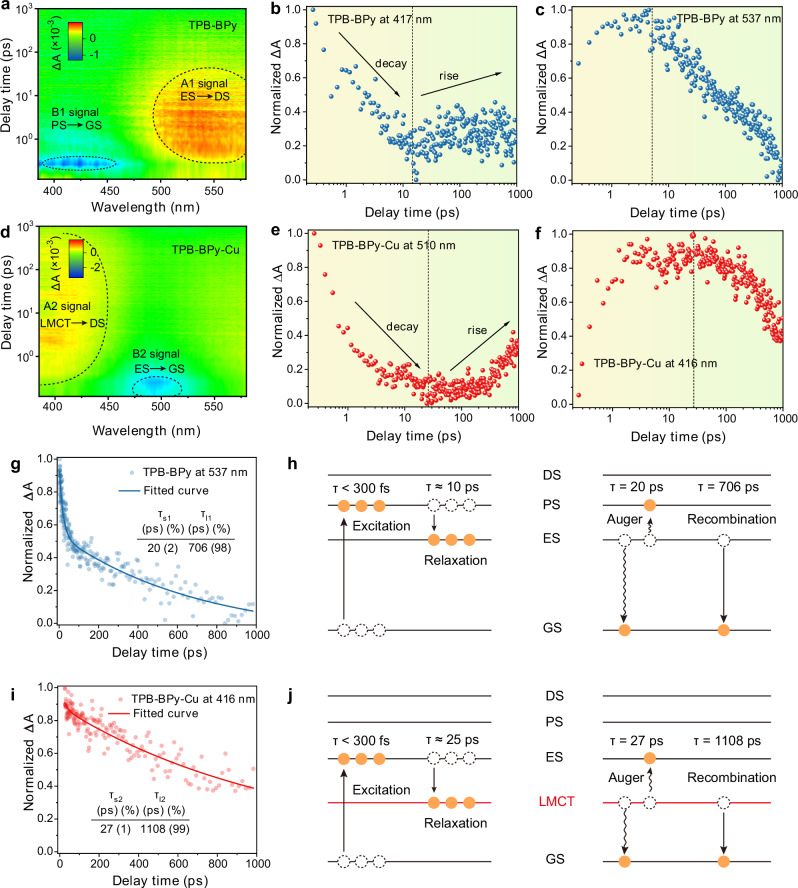
The transient behavior of excited states. **a** 2D fs-TA spectral mapping of TPB-BPy. Extracted decay kinetics of TPB-BPy at (**b**) *λ* = 417 and (**c**) *λ* = 537 nm, respectively. **d** 2D fs-TA spectral mapping of TPB-BPy-Cu. Extracted decay kinetics of TPB-BPy-Cu at (**e**) *λ* = 510 and (**f**) *λ* = 416 nm, respectively. **g** Normalized decay kinetic plots for TPB-BPy at 537 nm and (**h**) the fitted lifetimes of different transient behaviors. **i** Normalized decay kinetic plots for TPB-BPy-Cu at 416 nm and (**j**) the fitted lifetimes of different transient behaviors.

The photophysical process dynamics were elucidated using fs-TA spectroscopy performed on TPB-BPy, the photoinitiator in TPB-BPy-Cu^43–46^. As shown in Fig. 7a, the 2D fs-TA spectral mapping of TPB-BPy exhibits two signals. The negative bleaching signal (B1) around 417 nm arises from the relaxation of the PS, since its wavelength is close to the PL2 of TPB-BPy^47–49^. The positive excited-state absorption signal (A1) at 537 nm is attributed to a lower-to-higher excited state transition (Supplementary Fig. 10)^50^. Their kinetic profiles (Fig. 7b, c) reveal distinct behaviors: the B1 signal decays rapidly (within 15 ps) and then rises, whereas the A1 signal rises until ~6 ps before decaying. The different decay trends of B1 and A1 indicate that the two signals correspond to different excited states. Since the A1 signal is enhanced as the B1 signal decreases in the early delay time, it can be inferred that A1 corresponds to an excited state lower than PS, namely, the ES. At longer times, the increase of the B1 signal and the decay of the A1 signal indicate an Auger relaxation of excitons^51^. Based on the above analysis, the photophysical process of TPB-BPy involves excitation to its PS, relaxation to ES, and potential re-excitation to dissociated charge carriers (Supplementary Fig. 11a, b).

**Fig. 8:**
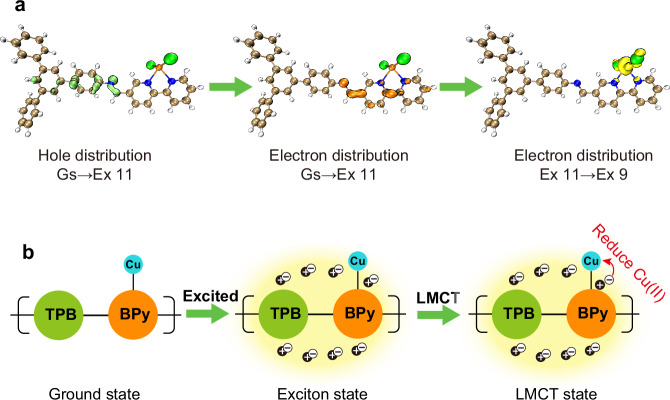
Simulated charge transfer behaviors. **a** Distribution of electron and hole isosurface over excited states No. 11 and 9 (isovalue = 0.005), which can be interpreted as the ES and LMCT states of TPB-BPy-Cu, respectively. **b** The transient behavior of excited states within TPB-BPy-Cu.

The fs-TA spectra of TPB-BPy-Cu (Fig. 7d) exhibit two absorption bands: B2 at ~500 nm (exciton relaxation) and A2 at 416 nm (lower-to-higher excited state transition). Their kinetic profiles (Fig. 7e, f) also reveal two phases: the B2 signal decays as the A2 signal intensifies at the early stage (<25 ps), and then the trend reverses (Supplementary Fig. 12). These results demonstrate that TPB-BPy-Cu undergoes similar photophysical processes as TPB-BPy: TPB-BPy-Cu is first excited to a middle-excited state (B2 signal), and then relaxes to a lower excited state, which can be re-excited to a higher energy level (A2 signal). However, unlike TPB-BPy with PS as its middle-excited state, the middle-excited state of TPB-BPy-Cu corresponds to ES. Hence, the lower excited states are interpreted as the LMCT states, and the intensity of the A2 signal can reflect the population of the LMCT states, i.e., the concentration of generated Cu(I) species. These results were verified by time-dependent DFT calculations^52,53^. As summarized in Supplementary Table 3, fifteen excited states were computationally screened, among which two dominant states (No. 11 and 9) exhibit the highest oscillator strengths. For excited state No. 11, the electrons and holes primarily localize on the ligand (Fig. 8a), which confirms its π/π* occupancy. Hence, excited state No. 11 corresponds to the ES state of TPB-BPy-Cu. In contrast, the electron distribution of excited state No. 9 shifts significantly toward the Cu center (Supplementary Table 4), while the holes remain on the ligand (Supplementary Fig. 13). This spatial migration confirms the LMCT process, as schematically illustrated in Fig. 8b. As such, the actual photophysical process can be described as follows (Supplementary Fig. 11c). Upon irradiation, TPB-BPy-Cu is excited to the ES and quickly relaxes to the LMCT state, which is manifested by the decay of the B2 signal and the enhancement of the A2 signal. On the prolonged time scale, the LMCT state relaxes to the GS, and the accompanying Auger recombination leads to restoration of the ES, thus resulting in the reverse trend of the B2 and A2 signals in the fs-TA spectra.

Analysis of the fs-TA spectra highlights the crucial role of the lowest excited states in generating active Cu(I) species due to their rapid formation and long lifetimes. Global fitting of the A1 and A2 signals reveals lifetimes for TPB-BPy (ES) and TPB-BPy-Cu (LMCT state)^54–56^. As shown in Fig. 7g, h, the A1 decay curve fits to a biexponential function: a fast pathway (*τ*_s1_ = 20 ps) for Auger recombination and a slower pathway (*τ*_l1_ = 706 ps) for radiative recombination, matching its fluorescence lifetime (*τ*_f1_ = 853 ps, Supplementary Fig. 14)^57^. Similarly, fitting of the A2 decay for TPB-BPy-Cu (Fig. 7i, j) shows short (*τ*_s2_ = 27 ps) and long (*τ*_l2_ = 1108 ps) lifetimes for Auger and radiative relaxation, respectively, and the long lifetime aligns with its fluorescence lifetime (*τ*_f2_ = 1078 ps). Notably, the addition of 10 mM benzylazide and phenylacetylene (substrates for the CuAAC reaction) slows the decay of the LMCT state (Supplementary Fig. 15), suggesting that the photogenerated Cu(I) species can be easily coupled to the substrates (Supplementary Fig. 16) and remain stable during the photocatalysis reaction.

### Photocatalytic CuAAC reaction over TPB-BPy-Cu

**Fig. 9:**
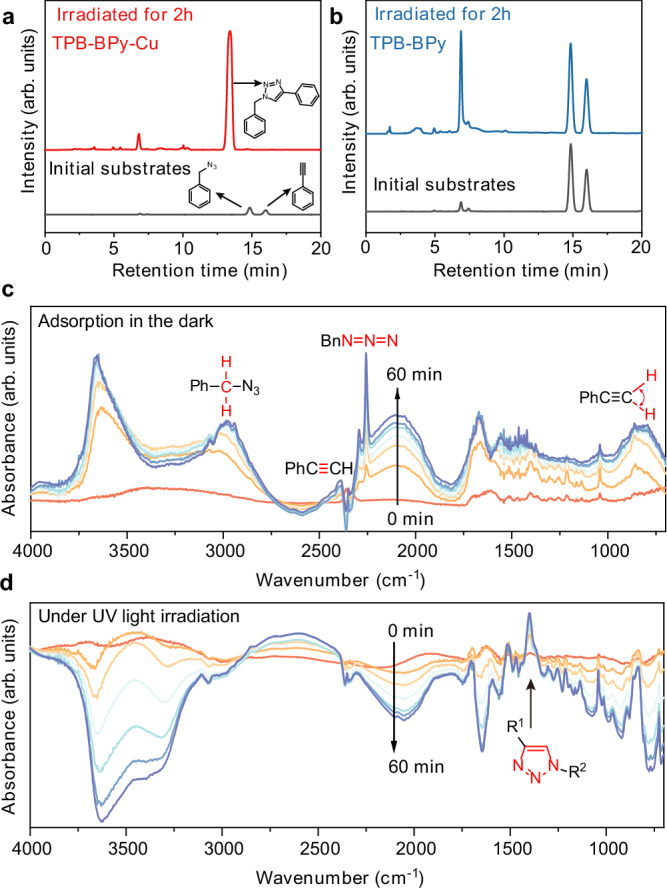
The photocatalytic processes and mechanisms. HPLC spectra for photocatalytic CuAAC over (**a**) TPB-BPy-Cu and (**b**) TPB-BPy. **c** Vibrational bands of substrates as they are adsorbed on the surface of TPB-BPy-Cu. **d** Vibrational signals of substrates, intermediates, and products upon light irradiation.

The rapid ligand→Cu electron transfer and prolonged lifetime of LMCT state suggest that the TPB-BPy-Cu complex could provide an efficient platform for light-triggered CuAAC click chemistry reaction without the need for any sacrificial electron donor. In accordance with our predictions, as a model reaction system, alkynes and azides could be efficiently coupled to form triazoles, as confirmed by ^1^H nuclear magnetic resonance (NMR) spectroscopy (Supplementary Figs. 17–23). As monitored by high-performance liquid chromatography (HPLC, Supplementary Fig. 24), TPB-BPy-Cu achieved 95% substrate conversion (for benzylazide and phenylacetylene) and >90% triazole yield (Fig. 9a and Supplementary Fig. 25) as well as high cycling stability (Supplementary Table 5) without the need for sacrificial agents. The turnover number (TON) and turnover frequency (TOF) are calculated to be 42.2 and 21.1 h^−1^, respectively. Besides, the single-atom catalyst exhibited a robust coordination structure after long-term light irradiation (Supplementary Fig. 26 and Supplementary Table 6). By contrast, TPB-BPy showed no activity (Fig. 9b). Adding Cu(II) salt to TPB-BPy yielded only 5% conversion (Supplementary Fig. 27), ascribed to the low efficiency of Cu(I) formation in a homogeneous system containing Cu(II). These results highlight the advantage of single-atom Cu-loaded COF in the photocatalytic click reaction.

There are two typical catalytic mechanisms for launching the CuAAC reaction at single catalytic sites (Supplementary Fig. 28), i.e., the formation of Cu acetylide (C ≡ C-Cu) or Cu-containing three-membered ring^58–60^. In situ diffuse-reflectance infrared Fourier transform spectroscopy (DRIFTS, Supplementary Fig. 29) was conducted to elucidate the reaction mechanism^61,62^. According to molecular vibration calculation (Supplementary Figs. 30–33), the band at ~2102 cm^−1^ is assigned to terminal C ≡ C-Cu vibration, indicating that the reaction proceeds via Cu acetylide formation (Fig. 9c). Moreover, a band at ~1401 cm^−1^ confirms the production of triazole (Fig. 9d). These results suggest that the reaction involves PhC≡C-CuL_n_ intermediate formation followed by [2 + 3] cycloaddition with azide.

## Discussion

This study presents a new paradigm in click chemistry using a recyclable single-atom Cu(II) photocatalyst. Firstly, the fine structure of TPB-BPy-Cu was characterized by XAS and XPS, confirming the single-atom dispersion of Cu(II) ions in COF. Spectral and DFT analyses revealed the involvement of LMCT during the photophysical process. Upon illumination, the electrons of TPB-BPy-Cu are excited from π to π* orbitals. Owing to the ligand field effect, the 3 *d* orbitals of Cu(II) split into five atomic orbitals, the highest of which lies between the π and π* orbitals and is occupied by an unpaired electron. Hence, the π* electron can easily inject into the split 3 *d* orbital of Cu, namely, the LMCT energy level. The ultrafast electron injection process was delineated by fs-TA spectroscopy. The lifetime of the LMCT state is 1108 ps, which is significantly longer than the lifetime of the exciton state of TPB-BPy. Benefiting from the fast generation of Cu(I) species and its long lifetime, TPB-BPy-Cu exhibits a high efficiency for photocatalytic coupling of benzylazide with phenylacetylene into triazoles, a model CuAAC reaction that can be induced by Cu(II) under the sole action of light. CuAAC is promoted by a short-lived transient Cu(I) without the need for external agents and returning to the initial ground state after carrying out the alkynyl-azide cycloaddition. Moreover, the cycloaddition route was elucidated by in situ DRIFTS, which showed the formation of PhC≡C-CuL_n_. These findings provide a new platform for efficient click chemistry reactions with spatial control exclusively within the irradiated zones and deepen our understanding of organic transformation reactions on single-atom photocatalysts.

## Methods

### Synthesis of BPy-Cu

An acetone solution (5 mL) of BPy (21.2 mg, 0.1 mmol) was dropwise added into an acetone solution (5 mL) of CuCl_2_·2H_2_O (17.1 mg, 0.1 mmol) under continuous stirring. A green solid gradually precipitated from the solution at room temperature. After stirring for 2 h, the precipitate was collected by centrifugation and washed with acetone (3 × 10 mL) to remove the residual Cu and bipyridine. The yield reached 90% (31.1 mg).

### Synthesis of COFs

The two COFs (i.e., TPB-BPy and TPB-BPy-Cu) were synthesized by the Schiff-base polycondensation method. In typical procedures, a mixture of amine and aldehyde monomers and solvents were added into a 50-mL Schlenk tube. The mixture was flash-frozen in a liquid nitrogen bath and protected with argon gas during three freeze-pump-inflate cycles. Then the tube was sealed and placed in an oven at 120 °C for 3 d. The resulting precipitate was isolated by centrifugation and washed with acetone (3 × 10 mL) and pure water (3 × 10 mL). The sample was then dried at 80 °C in a vacuum oven for 12 h. Information on the chemicals and recipe in the synthesis is provided in the Supplementary Information (SI).

### Characterization

The materials were characterized by using mass spectroscopy, XRD, FTIR, XPS, HAADF-STEM, XAS, UV-visible diffuse reflectance spectroscopy, ultraviolet photoelectron spectroscopy, PL, time-resolved PL, temperature-dependent PL, and fs-TA spectroscopy. More details on the characterization methods are provided in the SI.

### Photocatalytic CuAAC tests

The photocatalytic experiments were carried out in a multi-channel photochemical reaction system. The conversion process was monitored by HPLC. The photocatalytic product was identified by ^1^H NMR spectroscopy. More detailed methods are provided in the SI.

### Computational details

The crystalline structures of TPB-BPy and TPB-BPy-Cu COFs were refined using the Reflex module of Materials Studio 8.0. The optimized repeating fragments were extracted as the initial structures, and their DOS was calculated using the DMol3 module. The calculations on frequency, molecular orbitals, and NBO analysis were carried out through the Gaussian 16 program package. The CDA on the repeating fragments of TPB-BPy and TPB-BPy-Cu were realized by using the Multiwfn program. More details on the computational methods are provided in SI.

## Supplementary information

Supplementary Information

Peer Review file

## Source data

Source Data

## Data Availability

Data are available from the corresponding authors upon request. All data generated in this study are provided in the Source Data file. Source data are provided with this paper.
