# Supplementary material for: Single-atom photocatalyst for click reaction
Source: Nat Commun. 2026 Jun 10;17:7376. doi: 10.1038/s41467-026-74278-4 (PMC13402698; doi:10.1038/s41467-026-74278-4)
Supplement: Supplementary file 1 — Supplementary Information [file 41467_2026_74278_MOESM1_ESM.pdf]

Supplementary Information

Single-atom photocatalyst for click reaction

Chang Cheng[1], Bicheng Zhu[1], Yue Lin[2], Zhifeng Jiang[3,*], Guijie Liang[4], Chuanjia Jiang[5,*], Hermenegildo García[6,*] & Jiaguo Yu[1,*]

Supplementary Methods

Materials

2,2'-bipyridine-5,5'-dicarbaldehyde (BPy) (≥ 97%), 1,3,5-tris(4-aminophenyl)benzene (TPB, 98%), $CuCl_2 \cdot 2H_2O$ (analytical grade), benzylazide (analytical grade), ethyl azidoacetate (≥ 95.5%), 2-ethynylpyridine (≥ 98%), 3-ethynylthiophene (≥ 97%), and phenylacetylene (99.8%) were obtained from Aladdin Reagent Co. Ltd. Acetone, acetic acid, mesitylene, 1,4-dioxane, acetonitrile, petroleum ether, and dichloromethane were purchased from Sinopharm Chemical Reagent Co., Ltd. All of the above reagents were of analytical grade and used in their original state.

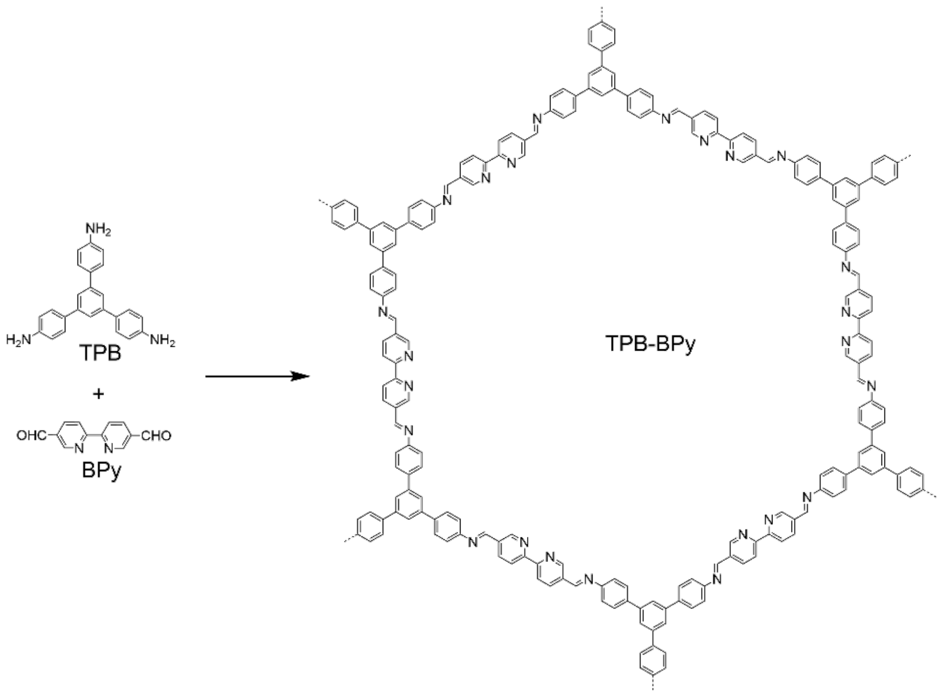

Synthesis of TPB-BPy. The BPy (19.8 mg, 0.09 mmol), TPB (21.1 mg, 0.06 mmol), and acetic acid (0.2 mL) were reacted in a solvent (6 mL, mesitylene:1,4-dioxane = 2:1) to produce metal-free TPB-BPy, with a yield of 89%.

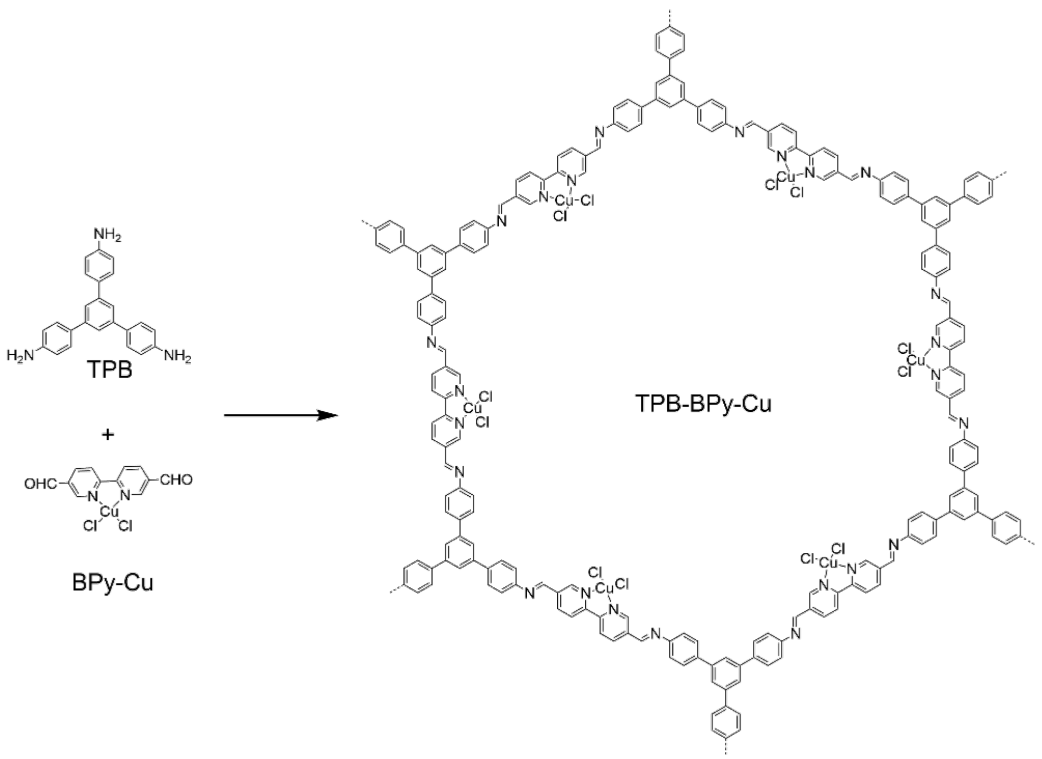

Synthesis of TPB-BPy-Cu. The Cu(II)-coordinated BPy (BPy-Cu) (31.1 mg, 0.09 mmol), TPB (21.1 mg, 0.06 mmol), and acetic acid (0.2 mL) were reacted in a solvent (6 mL, mesitylene:1,4-dioxane = 2:1) to produce TPB-BPy-Cu, with a yield of 81%.

Characterization. The ^{1}H NMR spectra were obtained on an Agilent 400-MR NMR spectrometer at room temperature. The mass spectrum of BPy-Cu was acquired on a RADIAN ASAP mass spectrometer (Waters, Singapore). A transmission electron microscope (Titan G2, FEI, USA) was used to perform morphology and microstructure observation. The XRD patterns of the samples were obtained on an X-ray diffractometer (D/Max-RB, Rigaku, Japan). The Fourier transform infrared spectroscopy and in situ DRIFTS were performed on a spectrometer (Nicolet iS50, Thermo Scientific, USA). The UV–vis diffuse reflectance spectra were obtained with a UV–vis spectrophotometer (UV2600, Shimadzu, Japan). The ultraviolet photoelectron spectroscopy (UPS) and in situ irradiated XPS analyses were performed on an electron spectrometer (ESCALAB 210, VG, UK) under light irradiation at λ = 365 nm. The actual content and leaching amount of Cu were determined by using an inductively coupled plasma optical emission spectrometer (ICP-OES, Ailent 720, USA). The time-resolved photoluminescence spectra were acquired using FLS1000 fluorescence lifetime

spectrophotometer (Edinburgh, Instruments, UK). The steady-state and temperature-dependent PL measurements were recorded on an FLS980 fluorescence lifetime spectrophotometer (Edinburgh, Instruments, UK). For steady-state PL measurements, the samples were dispersed in actonitrile. For temperature-dependent PL studies, the powder samples were directly probed and cooled by a 77 K nitrogen bath (OptistatDN, Oxford, UK) with a temperature controller (MercuryiTC, Oxford, UK). The exciton binding energy (E_b) can be derived by fitting the data to the Arrhenius equation (1):

$$I_{(T)} = \frac{I_{(0)}}{1 + A\exp(-\frac{E_b}{RT})} \quad (1)$$

where T is temperature, and $I_{(0)}$ and $I_{(T)}$ are the intensity at 0 K and temperature T, respectively. R is the gas constant. A is the pre-exponential factor.

X-ray absorption spectroscopy. X-ray absorption spectra were recorded at the Cu K-edge using a Table XAFS-500 instrument (Specreation Instruments, China). Cu foil was applied to calibrate the energy, and the data were collected in the absorption mode. The energy of the first maximum in the first derivative of the X-ray absorption near edge structure (XANES) was defined to be 8979 eV. The data subsequently underwent intensity normalization, baseline subtraction, and Fourier transformation using the Athena software. Then the $\mathbf{k}^2$-weighted R-space data were obtained. The amplitude reduction factor (S_0^2), coordination numbers (N), distances to the scattering atoms (R), and mean-squared displacements (σ^2) can be determined by fitting the R-space data using the Artemis program. The extended X-ray absorption fine structure (EXAFS) of individual paths were simulated by importing the modified XRD refinement results into Artemis and generating spectra using FEFF6. The in situ irradiated XANES and EXAFS spectra were recorded under LED irradiation (λ = 365 nm) on the sample cell, and the atmosphere was controlled by introducing high-purity nitrogen or benzylazide/phenylacetylene/acetonitrile vapor. The N K-edge soft X-ray absorption spectra (sXAS) were measured at the photoemission end-station at beamline BL10B (31131.02.HLS.PES) of the National Synchrotron Radiation Laboratory (NSRL) in Hefei, China. The in situ irradiated sXAS spectra were recorded under the irradiation through the window of the analysis chamber. The sXAS raw data were first calibrated from the Au 4*f* peak

as a standard and then processed by normalization procedures.

Transient absorption spectroscopy measurements. The fs-TA measurements were conducted on a pump-probe system. In detail, an 800-nm output pulse from a 1-kHz Ti:sapphire regenerative amplifier (Coherent) was split into two parts with a 50% beam splitter. One part was transmitted to an Optical Parametric Amplifier (TOPAS) to generate a pump beam and chopped by a synchronized chopper at 500 Hz. The other part was further split and transformed into white light, serving as the probe beam (less than 10% energy, 320-650 nm) and reference light. The time delay between the pump and probe beams was adjusted by a motorized optical delay line, and the absorbance change can be calculated with two adjacent probe pulses (pump-blocked and pump-unblocked). Samples were dispersed in pure acetonitrile (or a solution of the reaction substrates) at a concentration of 0.2 g L^{-1}. The mixtures were added into quartz cuvettes with a path length of 2 mm, and then the cuvettes were sealed using rubber septa caps and degassed with Ar for 10 min. All data were obtained using an excitation wavelength of 340 nm and optical power of 100 μW cm^{-2}.

The decay curves obtained from the fs-TA spectra were fitted by the following multi-exponential equation (2):

$$I_{(t)} = \mathrm{I}_{(0)} + \sum_{i=1}^{n} A_i \exp\left(-t/\tau_i\right) \qquad (2)$$

where $I_{(t)}$ and $I_{(0)}$ represent the transient signal intensity and baseline correction value, and t is the probe time delay. A_i and τ_i are amplitudes and decay times, respectively. The minimum number of components n to satisfactorily fit the experimental data is two.

Photocatalytic CuAAC tests. The photocatalytic experiments were carried out in a 60-mL gas-tight Pyrex flask. 2 mg of photocatalysts, 0.1 mmol of azide, 0.1 mmol of alkyne, and TEA (0.04 eq) were added in 10 mL of acetonitrile. The airtight system was completely evacuated using a vacuum pump. Then ~100 kPa high-purity Ar gas was injected. The solution was first stirred for 1 h to reach adsorption equilibrium and then irradiated with an LED light (PCX50C, Beijing Perfectlight Technology Co., Ltd, 420 nm, 3 W). The system was kept at a constant temperature (20 °C) by flowing cooling water. Noteworthily, for comparison, the photocatalytic

experiments of TPB-BPy were performed with and without $CuCl_2 \cdot 2H_2O$ (0.5 mM), respectively. The conversion process was monitored by HPLC (LC-20AD, Shimadzu, Japan), equipped with a UV-Vis detector (SPD-20A) and a C18 column (5 μm, 250 × 4.6 mm). The mobile phase was acetone/H_2O (v/v = 9:1) with a flow rate of 1 mL min^{-1}. The column temperature was set as 40 °C, and the detection wavelength was 260 nm. The pure product was obtained by silica gel chromatography using a petroleum ether/dichloromethane mixture (10:1) as the eluent and identified by ^1H NMR spectroscopy. The turnover number (TON) and turnover frequency (TOF) were calculated by the following equations (3 and 4):

$$\mathrm{TON} = \frac{\text{Number of converted molecules}}{\text{Number of active sites}} \quad (3)$$

$$\mathrm{TOF} = \frac{\mathrm{TON}}{\text{Reaction time}} \quad (4)$$

Notably, the number of active sites is estimated from the total amount of Cu single atoms in the reaction system, which leads to a conservative estimation (i.e., underestimation) of the TON and TOF values, since only the fraction of Cu atoms that are photoreduced to Cu(I) serve as the active sites for CuAAC reaction. According to ICP-OES test results, the actual content (in mass percentage) of Cu in TPB-BPy-Cu is 7.15%. Hence, there are 0.143 mg of Cu atoms in 2 mg of samples, namely, the number of active sites is 2.25 μmol.

Computational details. The crystalline structures of TPB-BPy and TPB-BPy-Cu COFs were refined using the Reflex module of Materials Studio 8.0. The optimized repeating fragments were extrated as the initial structures, and their DOS was calculated using the DMol3 module. The exchange–correlation interaction was described by generalized gradient approximation with the PBE functional. The energy cutoff was set as 450 eV. The convergence threshold in geometry optimization was set as 10^{-5} eV for energy and 0.05 eV $Å^{-1}$ for force.

The calculations on frequency, molecular orbitals, NBO analysis were carried out through the Gaussian 16 program package at the level of PBE1PBE/D3(BJ)/DEF2SVP. The excited states was calculated at the level of PBE1PBE/D3(BJ)/DEF2TZVP. The charge decomposition analyses on the repeating fragments of TPB-BPy and TPB-BPy-Cu were realized by using the Multiwfn program.

Supplementary Figures

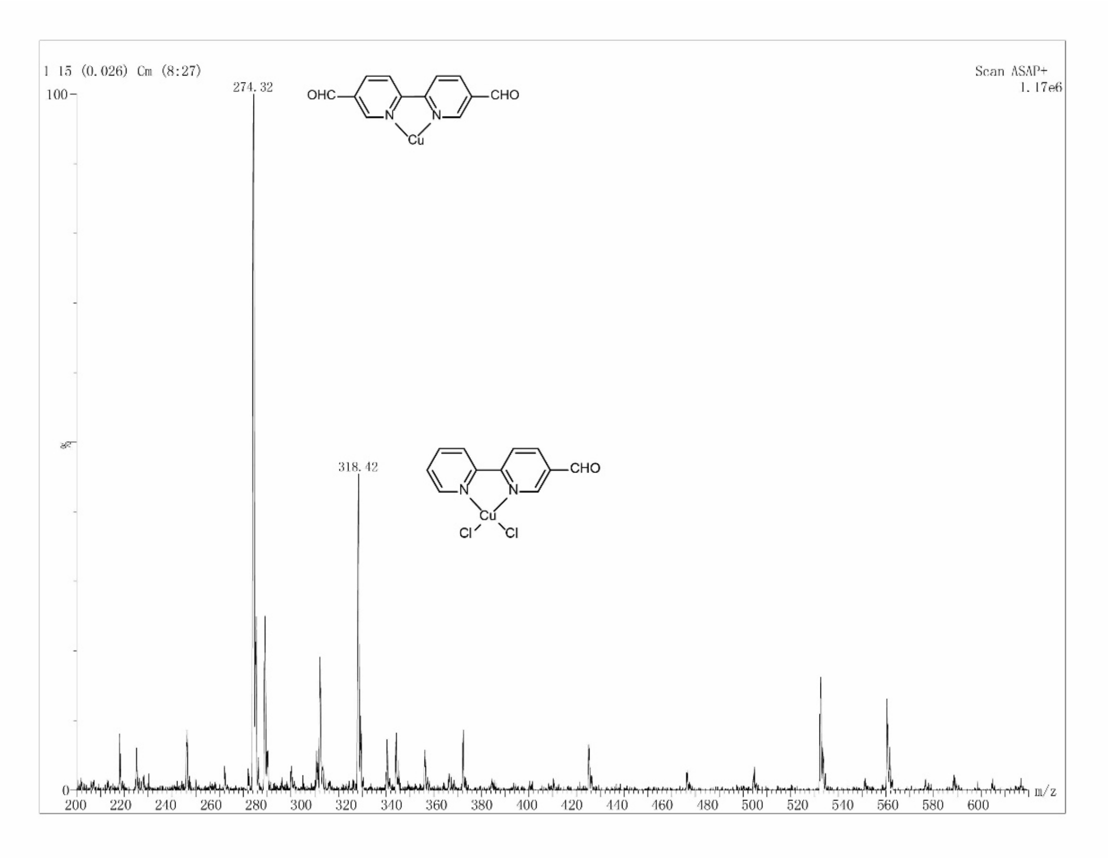

Supplementary Figure 1. The positive-ion mode mass spectrum of BPy-Cu monomer. The peak at *m*/*z* = 274.32 is assigned to $[Cu(BPy)]^+$, and that at *m*/*z* = 318.42 is attributed to $[CuCl_2(BPy)\text{-}CHO+H]^+$.

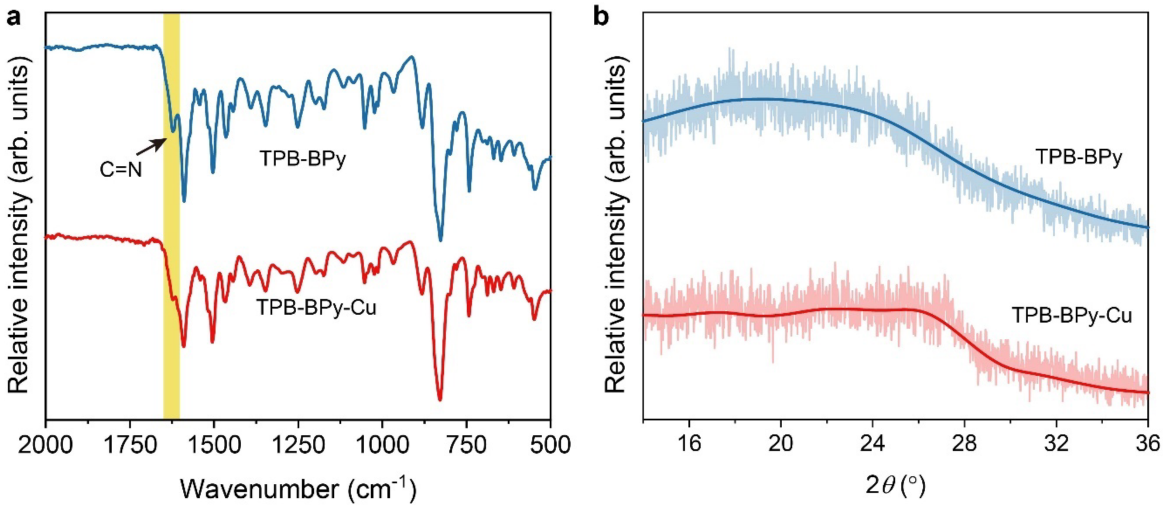

Supplementary Figure 2. Structure characterizations. a The Fourier transform infrared spectra of TPB-BPy and TPB-BPy-Cu. Both samples exhibit distinct C=N streching band at 1650 cm^{-1}, and no C=O stretching (~1700 cm^{-1}) signal is observed. **b** Powder X-ray diffraction patterns of TPB-BPy and TPB-BPy-Cu COFs. The hump peak at $2\theta \approx 20°$ is assigned to the intermolecular π-π stacking signal. According to Bragg's Law, the π-π stacking distance of TPB-BPy and TPB-BPy-Cu are calculated to be 4.55 and 3.41 Å, respectively.

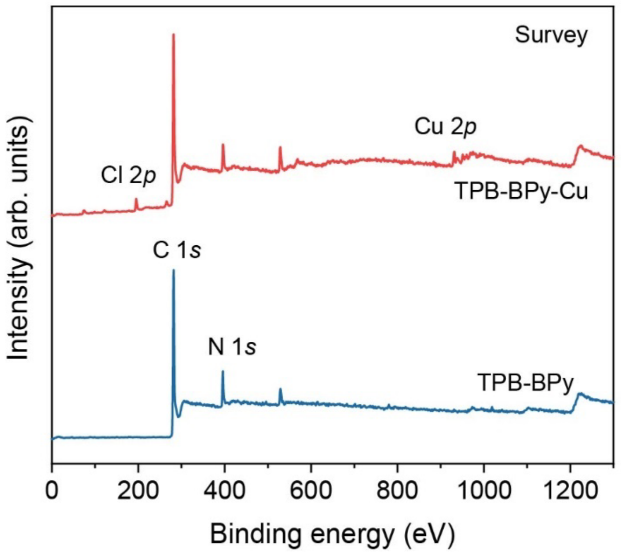

Supplementary Figure 3. XPS survey spectra of metal-free TPB-BPy and TPB-BPy-Cu. The atomic ratio of Cu/Cl/N is 1:2:4 over TPB-BPy-Cu, which is well in line with its theoretical ratio.

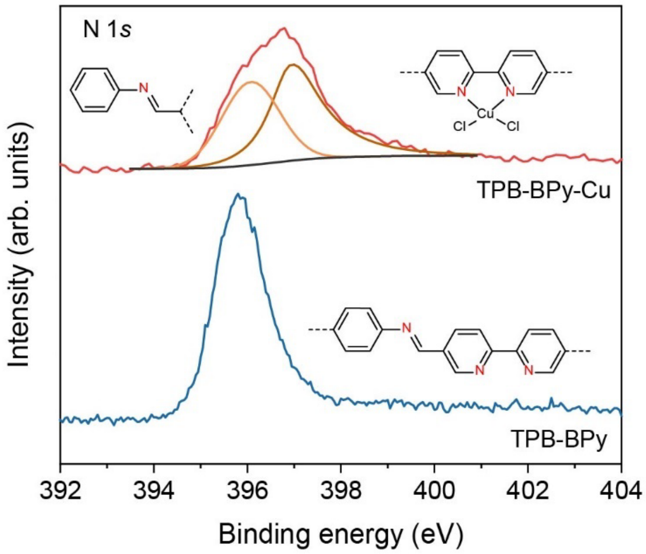

Supplementary Figure 4. High-resolution XPS spectra of N 1*s* in metal-free TPB-BPy and TPB-BPy-Cu. In TPB-BPy, the N 1*s* peak at 399.2 eV corresponds to imide and bipyridine N atoms, while in TPB-BPy-Cu, the bipyridine N peak is positively shifted to 400.4 eV. Both the imide N and bipyridinc N are sp^2 hybridized, which have similar chemical environment. Upon the coordination of Cu, the electron density of bipyridinc N is decreased (N→Cu), and accordingly the binding energy of bipyridinc N 1*s* core level increases. The imide/bipyridine N ratio (~1:1) aligns with the theoretical value, confirming Cu coordination at the bipyridine moiety.

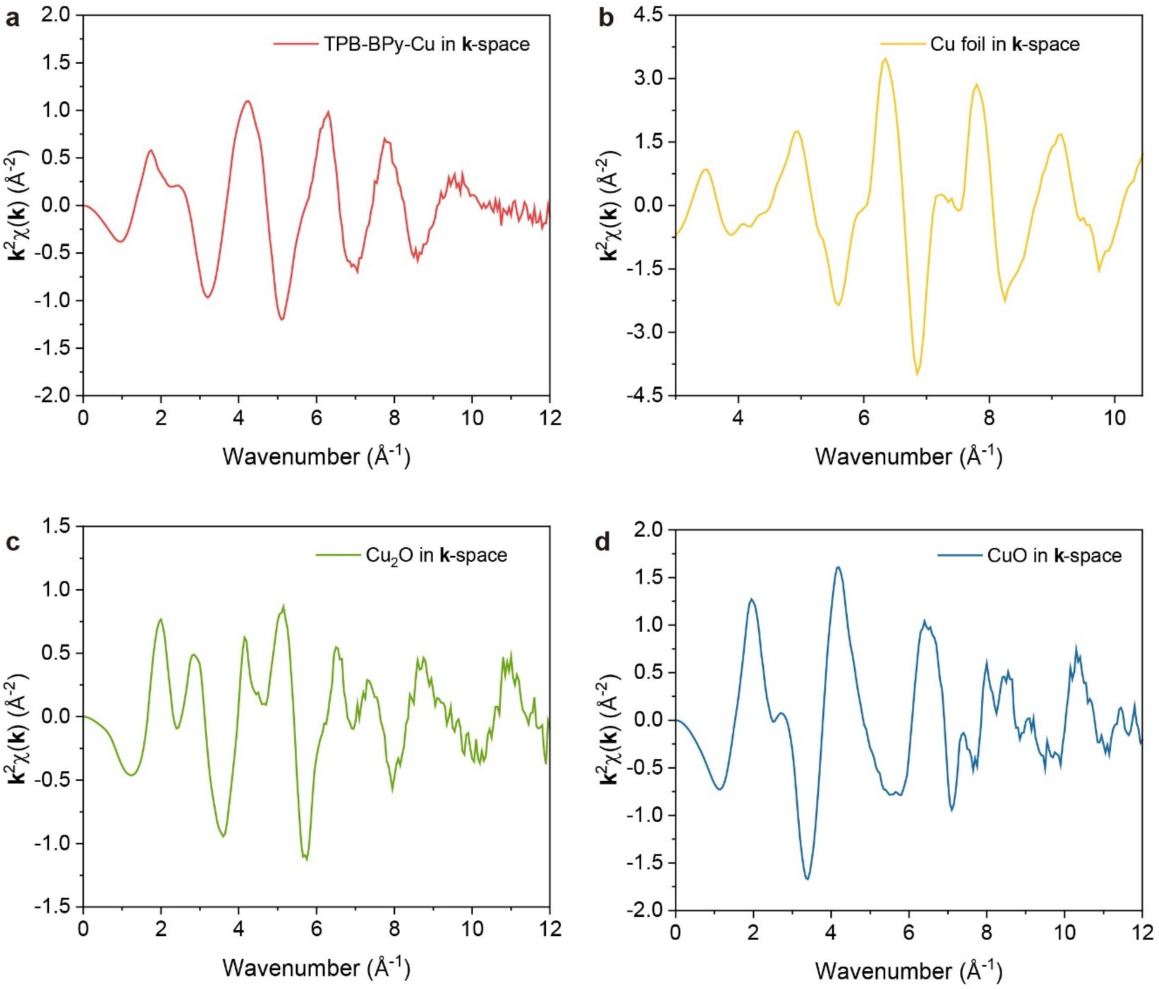

Supplementary Figure 5. Cu K-edge EXAFS for (**a**) TPB-BPy-Cu, (**b**) Cu foil, (**c**) Cu_2O, and (**d**) CuO in **k**^{2}-weighted **k**-space.

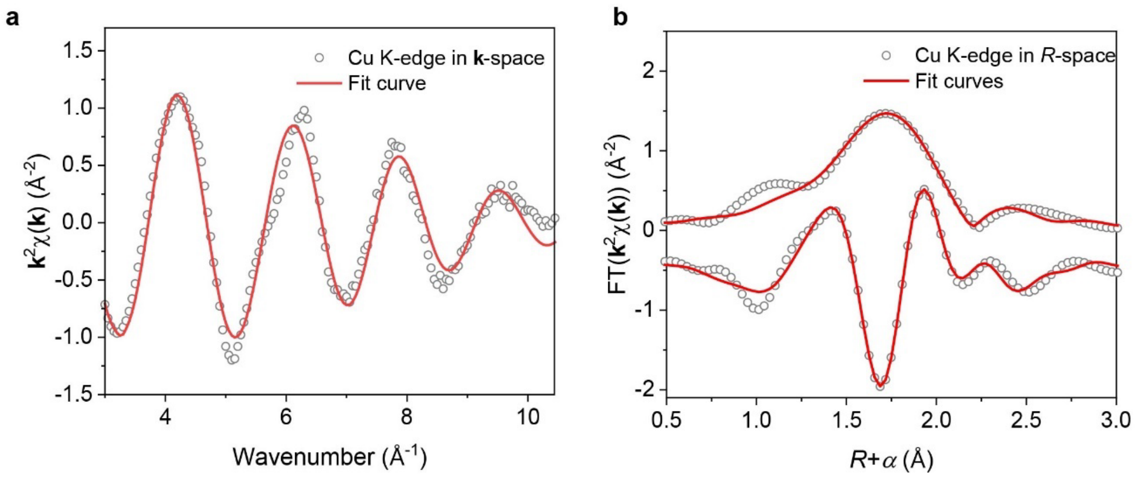

Supplementary Figure 6. Cu K-edge EXAFS spectrum for TPB-BPy-Cu in k^2-weighted (**a**) **k**-space and (**b**) *R*-space.

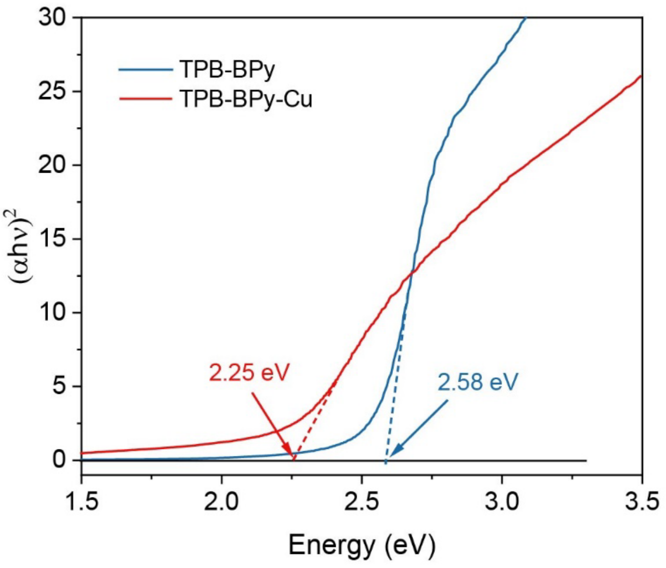

Supplementary Figure 7. Tauc plots of metal-free TPB-BPy and TPB-BPy-Cu samples. The fully conjugated molecular structure endows metal-free TPB-BPy with a moderate band gap of 2.58 eV. Upon coordination of Cu, the band gap of TPB-BPy-Cu is narrowed to 2.25 eV.

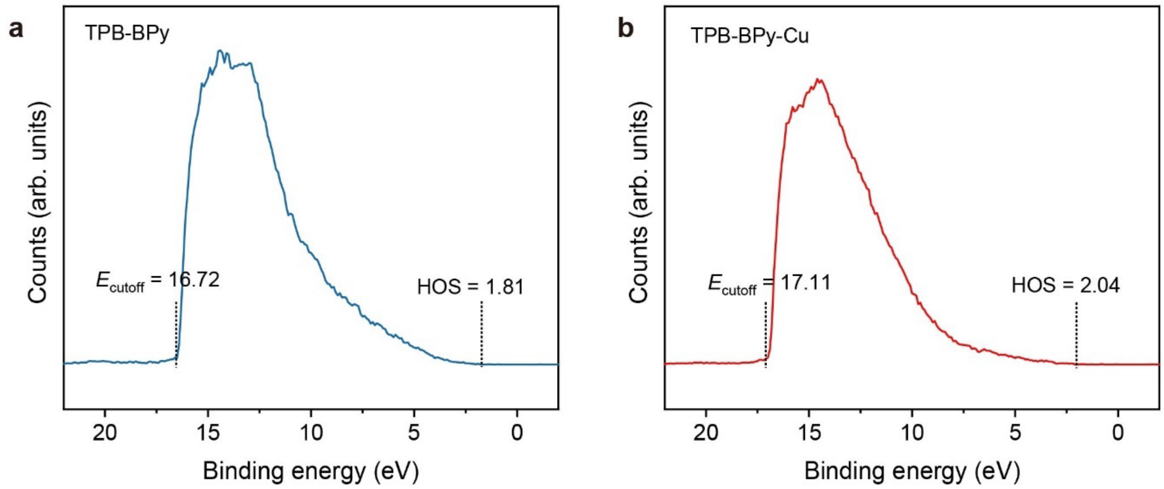

Supplementary Figure 8. Ultraviolet photoelectron spectra of (**a**) metal-free TPB-BPy and (**b**) TPB-BPy-Cu. According to the values of cutoff energy (E_{cutoff}) and the highest occupied states (HOS), ionization potential (Ip, equivalent to the negative value of the HOMO level vs. vacuum) can be obtained using the equation: $Ip = 21.22\ \mathrm{eV} - (E_{cutoff} - \mathrm{HOS})$. Therefore, the HOMO levels of metal-free TPB-BPy and TPB-BPy-Cu were calculated to be −6.31 and −6.13 eV, respectively.

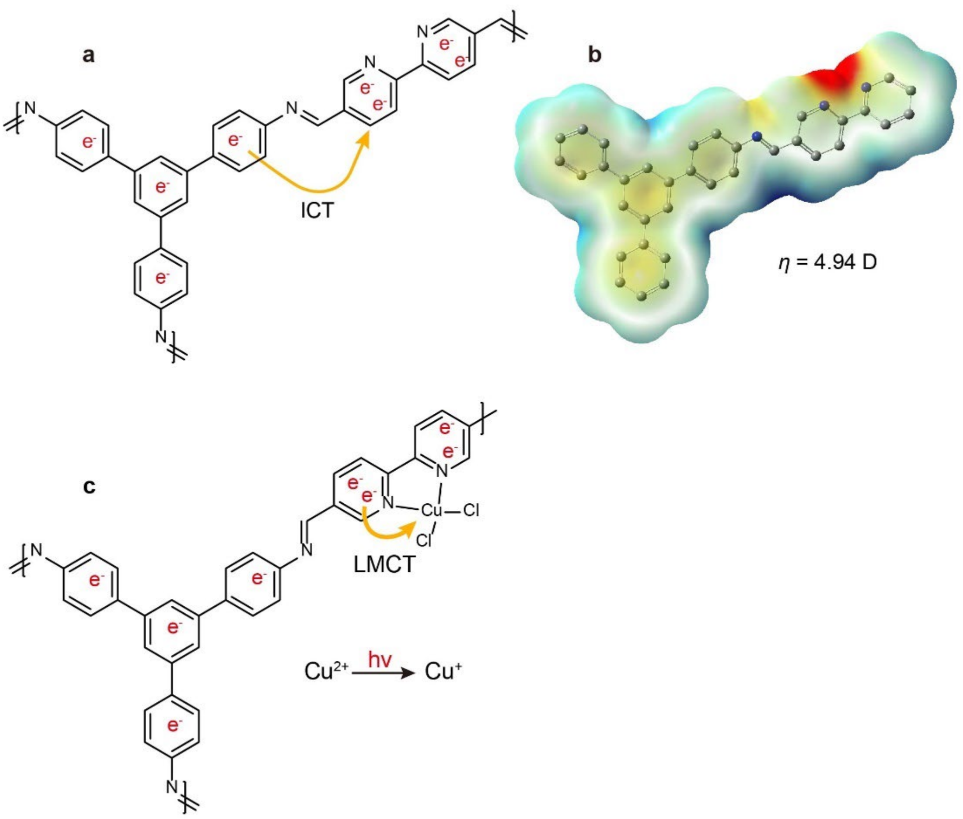

Supplementary Figure 9. Effect of molecular dipole moment. a Intramolecular charge transfer (ICT) within metal-free TPB-BPy units. **b** Electrostatic potential distribution and dipole moment (η) of TPB-BPy according to theoretical calculations. **c** Schematic illustration of the LMCT process.

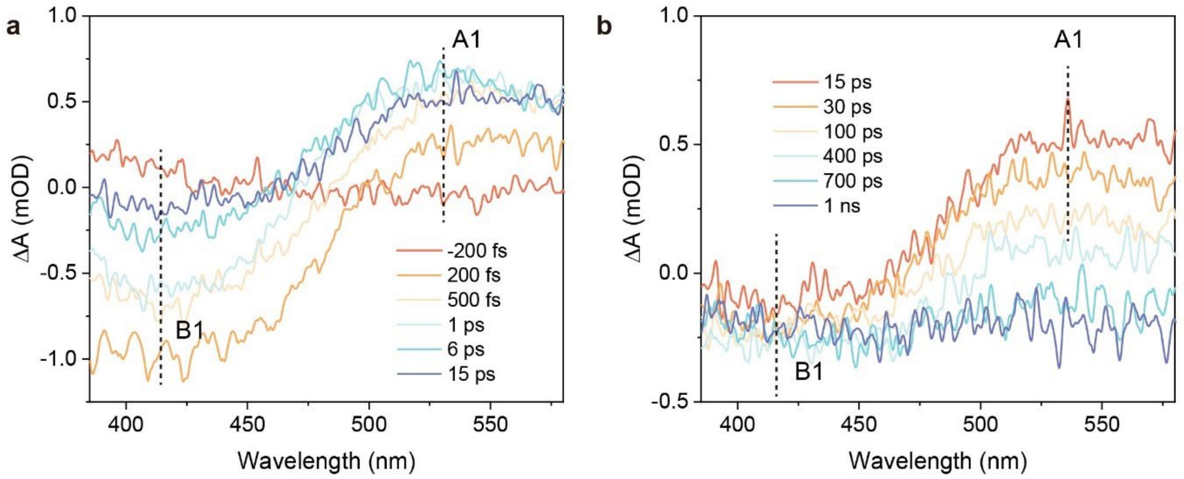

Supplementary Figure 10. Representative transient absorption spectra of TPB-BPy. The change in absorbance (ΔA) is defined as the optical density (OD) difference. The B1 signal decays before 15 ps (**a**) and then tends to rise (**b**), while the A1 signal first rises and then tends to decay.

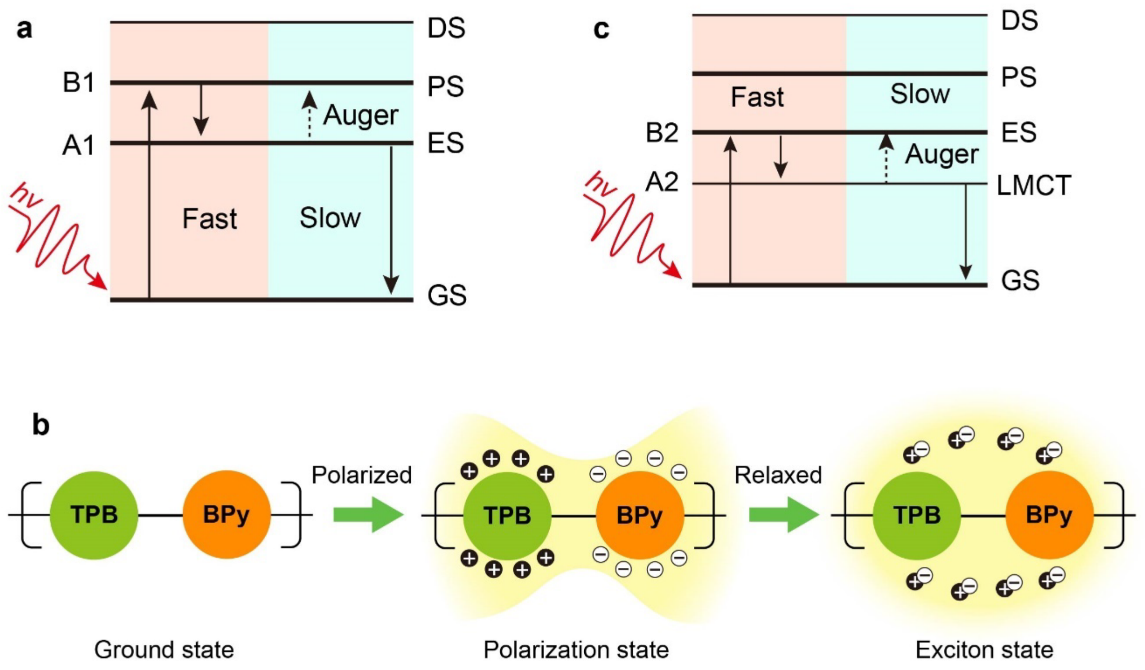

Supplementary Figure 11. Schematics of the transient behavior of excited states for (**a**, **b**) TPB-BPy and (**c**) TPB-BPy-Cu.

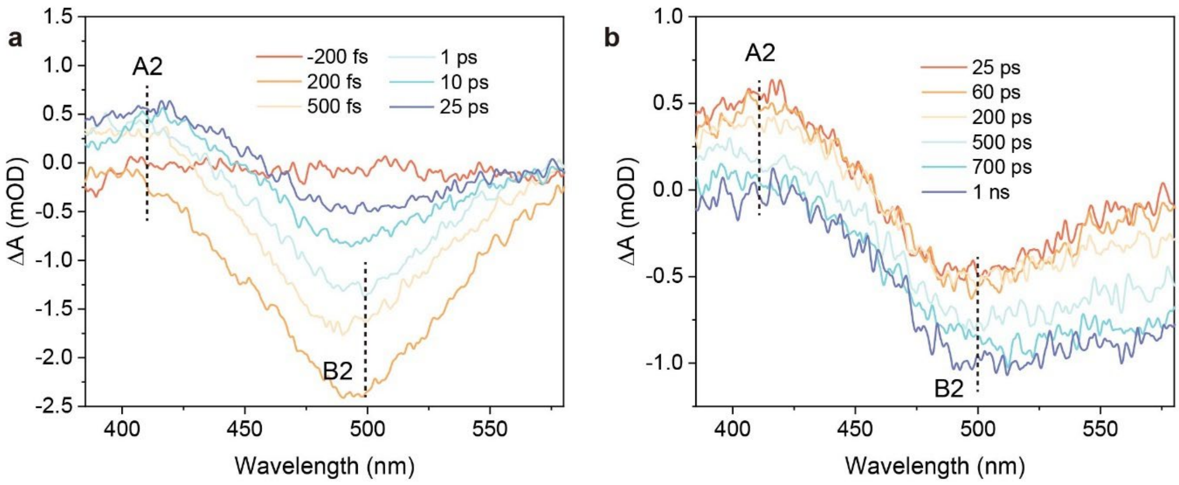

Supplementary Figure 12. Representative transient absorption spectra of TPB-BPy-Cu. The B2 signal decays before 25 ps (**a**) and then tends to rise (**b**), while the A2 signal first rises and then tends to decay.

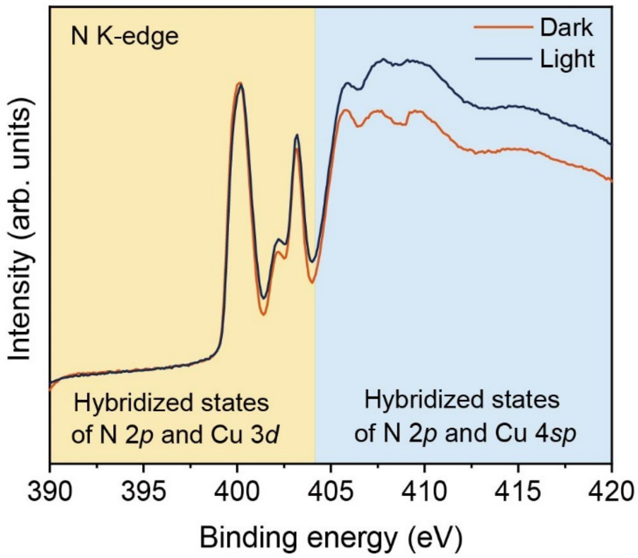

Supplementary Figure 13. In situ irradiated N K-edge sXAS analysis of TPB-BPy-Cu. The N *K*-edge XANES of TPB-BPy-Cu can be divided into two energy regions. The region from 390 to 404 eV is assigned to the N 1*s* → N(2*p*)-Cu(3*d*) transition. Theoretically, illumination should have little effect on the intensity of this transition because photoinduced LMCT occurs only between the N 2*p* and Cu 3*d* orbitals. Therefore, we normalized the peak in this region as a reference. The region from 404 to 420 eV is assigned to the N 1*s* → N(2*p*)-Cu(4*sp*) transition, where the contribution of Cu 4*sp* can be neglected because the photogenerated electrons are rarely injected into this orbital; thus, the absorption intensity is directly associated with the density of unoccupied N orbitals. Upon light irradiation, the absorption intensity in the high-energy region is distinctly enhanced, suggesting that a fraction of the N orbitals becomes unoccupied, which provides direct evidence for the generation of holes.

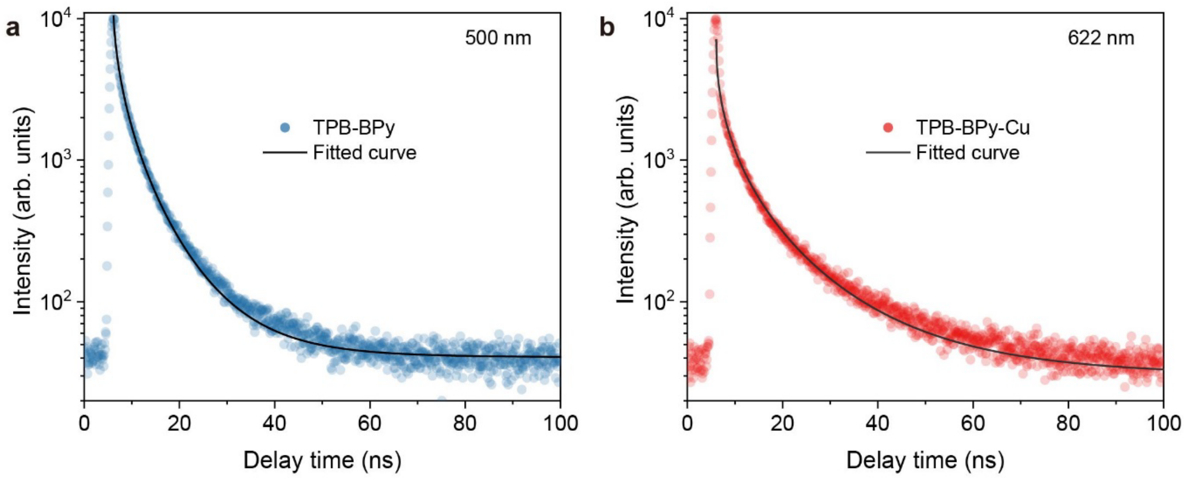

Supplementary Figure 14. Time-resolved fluorescence spectra of (**a**) metal-free TPB-BPy and (**b**) TPB-BPy-Cu. The decay curves obtained from the TRPL can be well-fitted by the following stretched exponential equation (5):

$$I_{(t)} = I_{(0)} + A_0 \exp\left(-(t/\tau_0)^{\beta_0}\right) \quad (5)$$

where $I_{(0)}$ and $I_{(t)}$ represent the baseline correction value and transient signal intensity, t is the delay time, A_0 is the pre-exponential factor, β_0 is the stretching exponent, and τ_0 represents the lifetime for exciton state of TPB-BPy (τ_{f1}) and trap state of TPB-BPy-Cu (τ_{f2}).

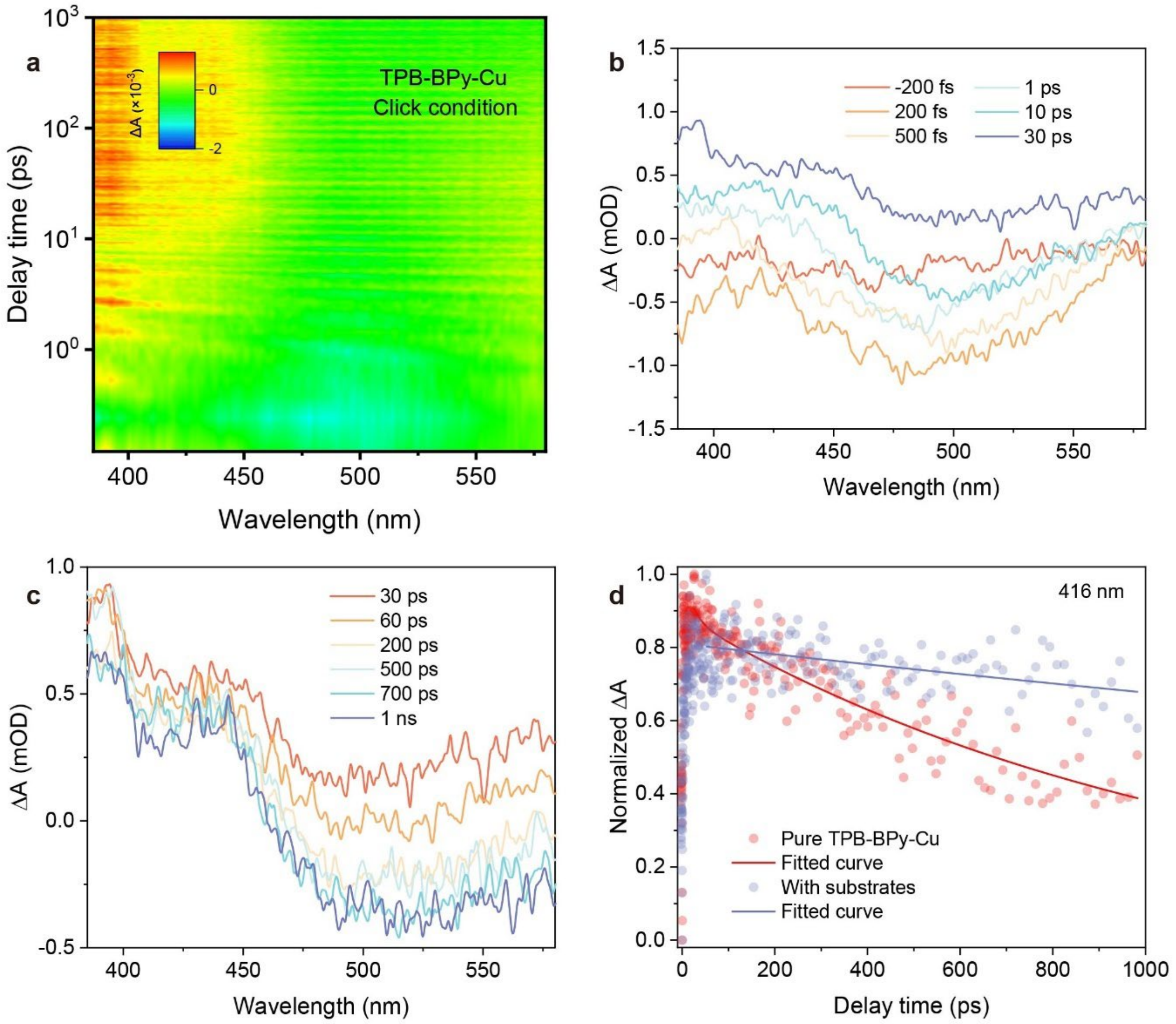

Supplementary Figure 15. TA spectra analysis of TPB-BPy-Cu in the presence of 10 mM benzylazide and phenylacetylene. **a** Two-dimensional TA spectral mapping. **b, c** Representative TA spectra in different time scale. (**d**) Comparison of the decay kinetics of TPB-BPy-Cu with or without substrates at λ = 416 nm. In the presence of substrates, the decay kinetics at λ = 416 nm, which reflects the population of the LMCT state, is slower than that in the absence of the substrates.

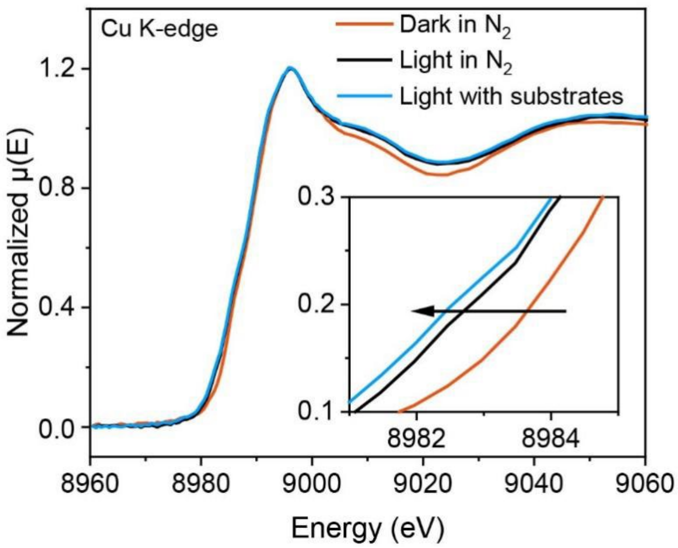

Supplementary Figure 16. In situ irradiated Cu K-edge XANES spectra of TPB-BPy-Cu under an atmosphere of N_2 or vapor of the substrates. Under N_2 atmosphere, the Cu K-edge negatively shifted upon light illumination, and it further negatively shifted under an atmosphere of benzylazide/phenylacetylene/acetonitrile vapor, suggesting interaction between the photogenerated Cu(I) species and the substrates.

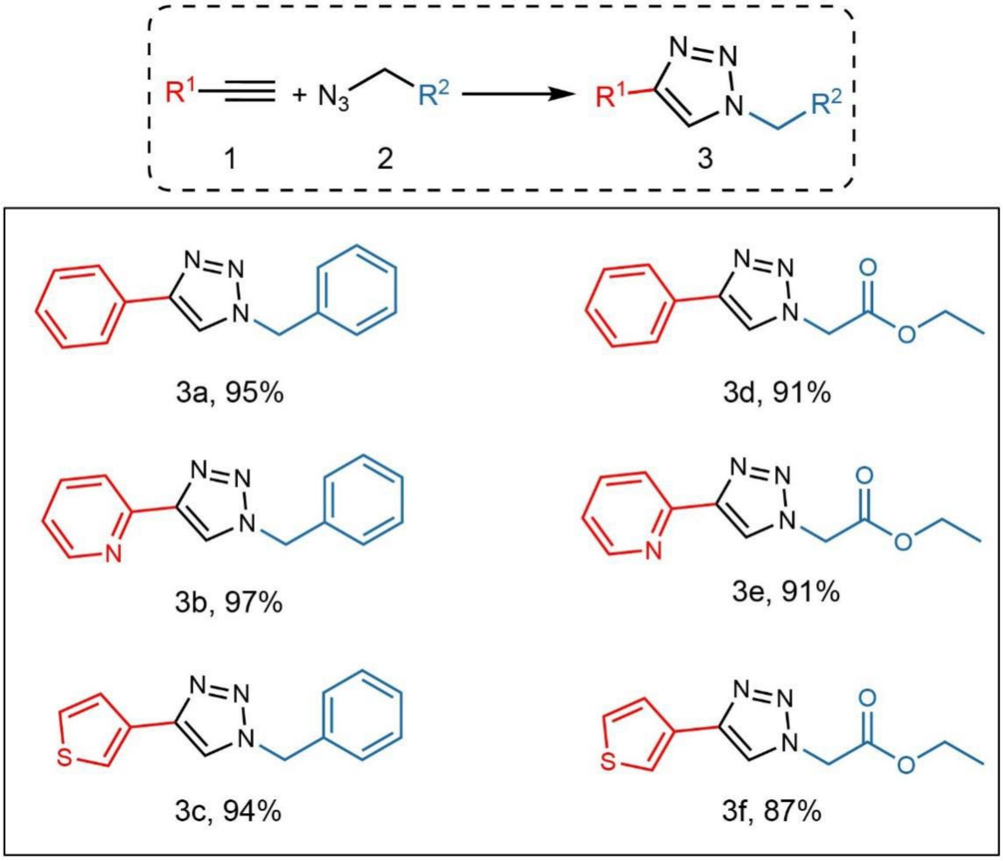

Supplementary Figure 17. Schematics for photocatalytic CuAAC reaction with different substrates and the yields of different products (3a to 3f) under the same condition. The alkynes containing electron-withdrawing (pyridine) and electron-donating (thiophene) groups also exhibited high reactivity. On the other hand, both aromatic and chain-like azides achieved excellent yields for the corresponding triazoles.

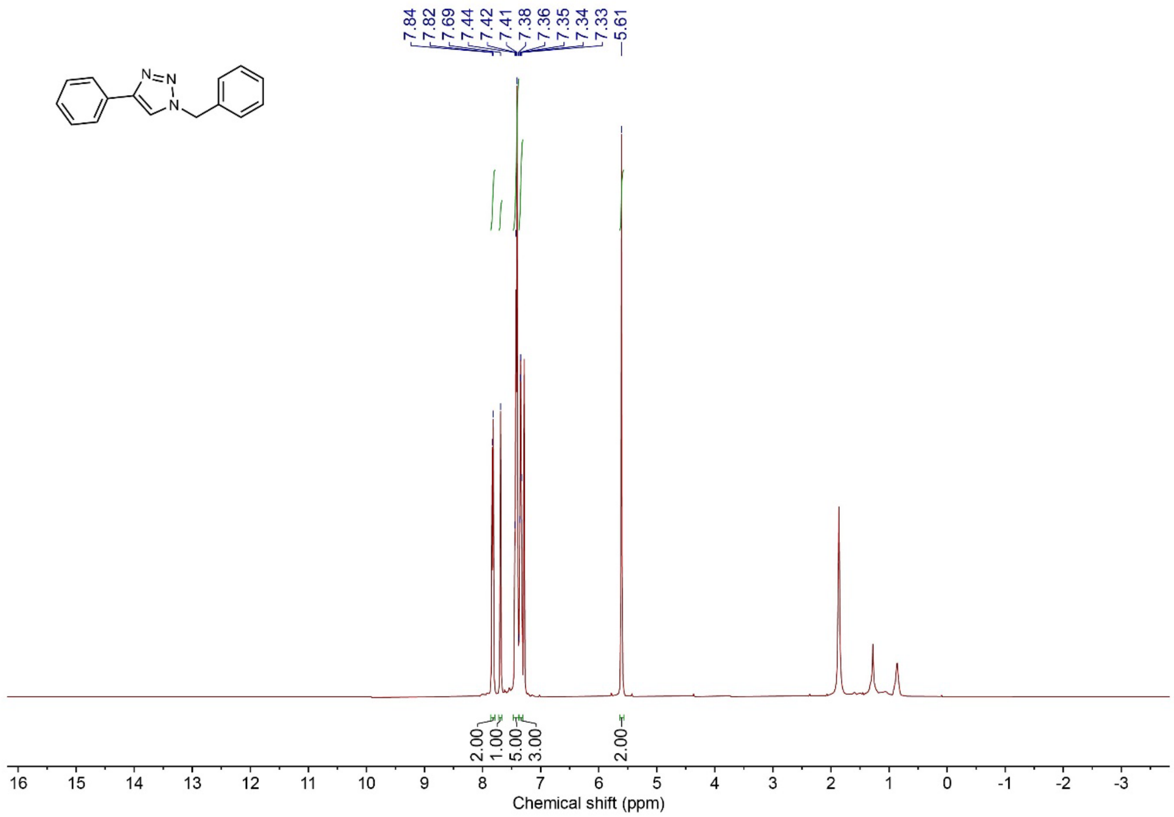

Supplementary Figure 18. The ^{1}H NMR spectrum of photocatalytic product 3a. The ^{1}H NMR (400 MHz, $CDCl_3$) chemical shifts at 7.83 (d, *J* = 7.5 Hz, 2H), 7.69 (s, 1H), 7.47–7.38 (m, 5H), 7.35 (q, *J* = 5.3, 4.6 Hz, 3H), and 5.61 (s, 2H) ppm are attributed to 3a,

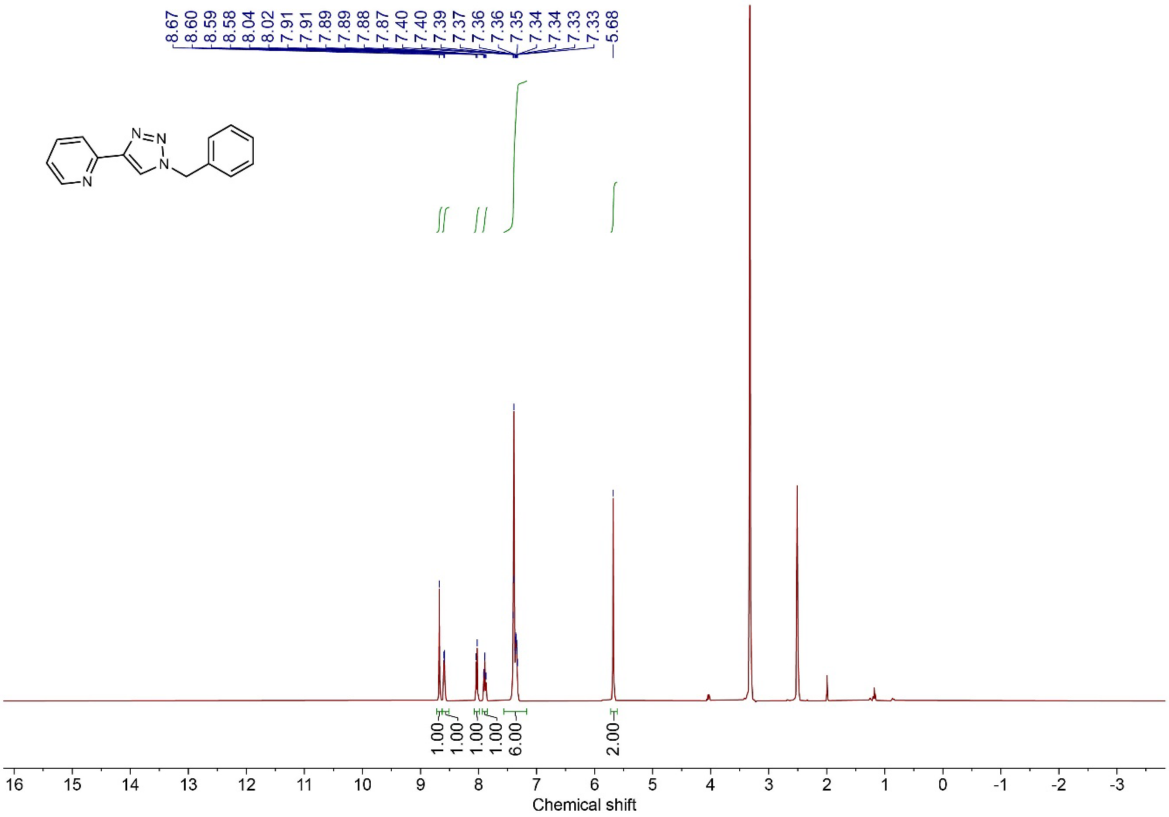

Supplementary Figure 19. The ^{1}H NMR spectrum of photocatalytic product 3b. The ^{1}H NMR (400 MHz, DMSO-d_6) chemical shifts at 8.67 (s, 1H), 8.59 (d, J = 4.3 Hz, 1H), 8.03 (d, J = 7.9 Hz, 1H), 7.89 (td, J = 7.8, 1.8 Hz, 1H), 7.41–7.31 (m, 6H), and 5.68 (s, 2H) ppm are attributed to 3b.

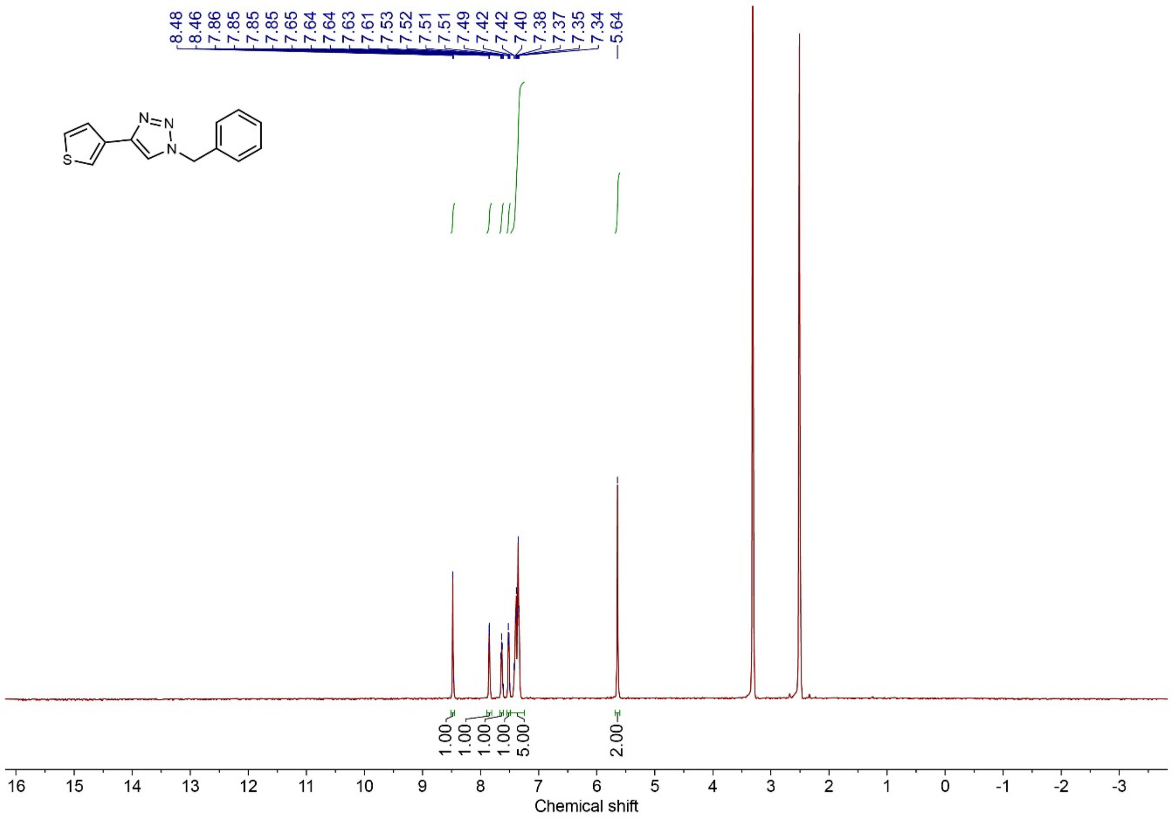

Supplementary Figure 20. The ^{1}H NMR spectrum of photocatalytic product 3c. The ^{1}H NMR (400 MHz, DMSO-d_6) chemical shifts at 8.48 (s, 1H), 7.85 (dd, J = 3.0, 1.5 Hz, 1H), 7.64 (dd, J = 5.0, 2.9 Hz, 1H), 7.52 (dd, J = 5.1, 1.4 Hz, 1H), 7.42–7.31 (m, 5H), and 5.64 (s, 2H) ppm are attributed to 3c.

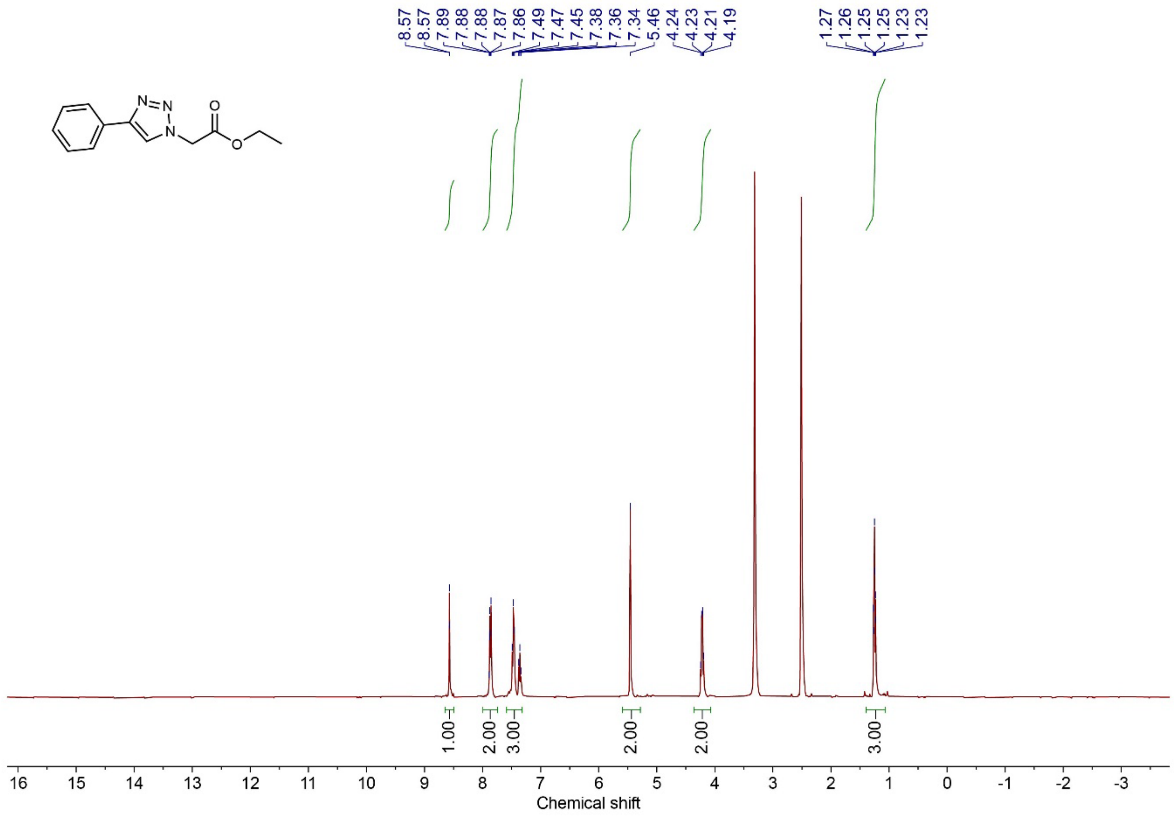

Supplementary Figure 21. The ^{1}H NMR spectrum of photocatalytic product 3d. The ^{1}H NMR (400 MHz, DMSO-d_6) chemical shifts at 8.57 (s, 1H), 7.87 (d, J = 7.7 Hz, 2H), 7.41 (dt, J = 45.1, 7.5 Hz, 3H), 5.46 (s, 2H), 4.22 (q, J = 7.0 Hz, 2H), and 1.25 (t, J = 7.1 Hz, 3H) ppm are attributed to 3d.

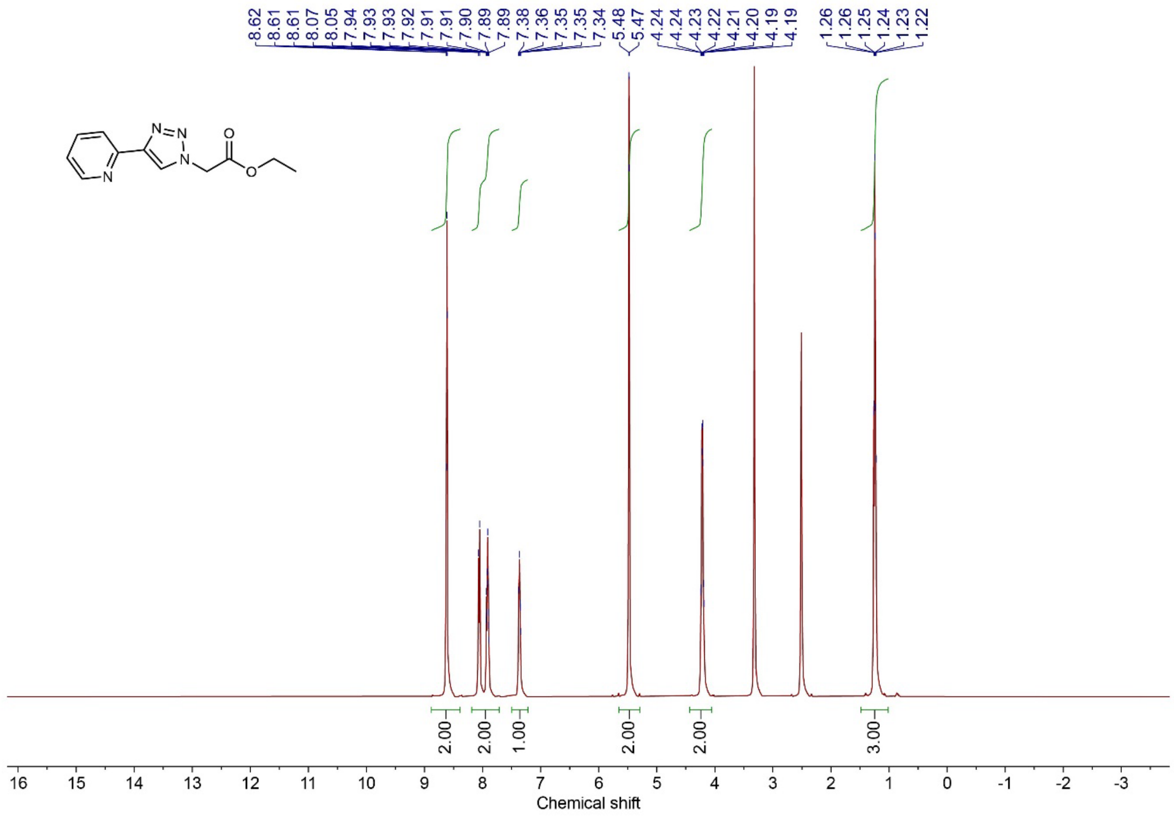

Supplementary Figure 22. The ^{1}H NMR spectrum of photocatalytic product 3e. The ^{1}H NMR (400 MHz, DMSO-d_6) chemical shifts at 8.62 (t, J = 3.2 Hz, 2H), 8.07–7.89 (m, 2H), 7.36 (t, J = 6.2 Hz, 1H), 5.48 (d, J = 2.1 Hz, 2H), 4.21 (qd, J = 7.1, 2.2 Hz, 2H), and 1.24 (td, J = 7.1, 2.2 Hz, 3H) ppm are attributed to 3e.

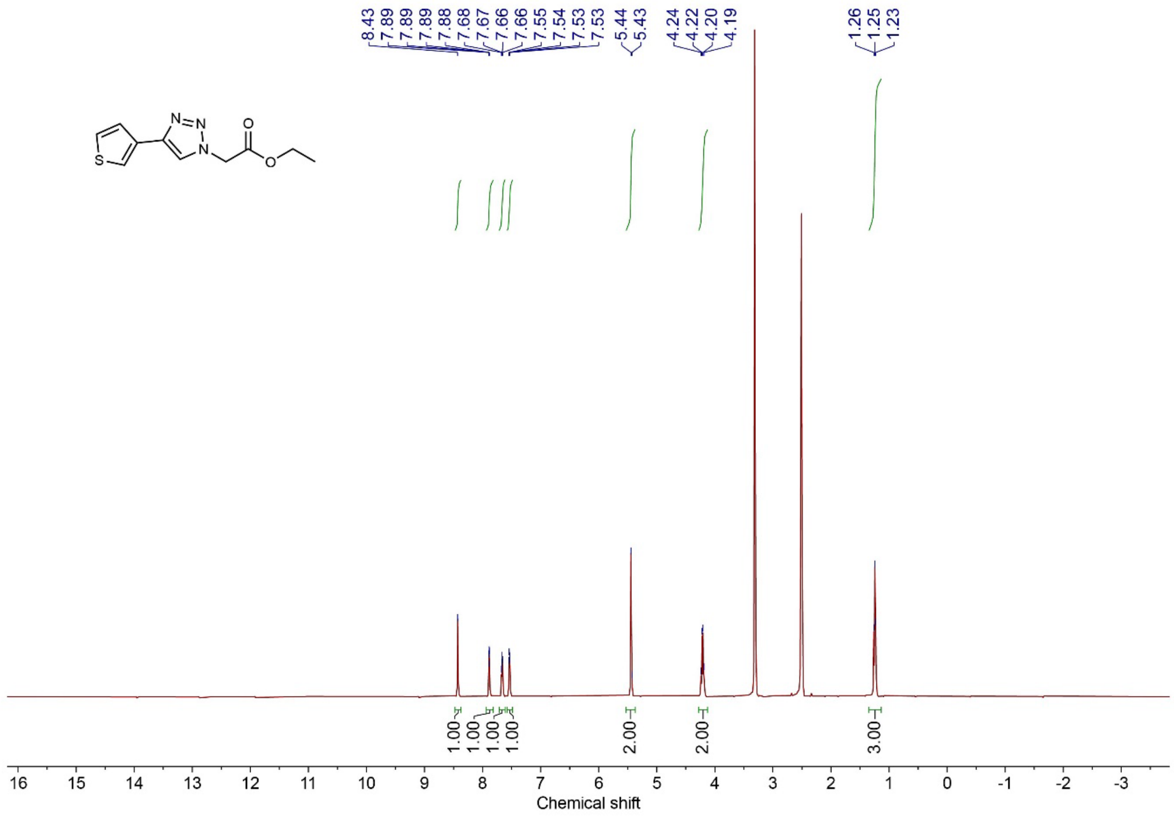

Supplementary Figure 23. The ^{1}H NMR spectrum of photocatalytic product 3f. The ^{1}H NMR (400 MHz, DMSO-d_6) chemical shifts at 8.43 (s, 1H), 7.89 (dd, J = 3.0, 1.3 Hz, 1H), 7.67 (dd, J = 5.1, 2.9 Hz, 1H), 7.55–7.53 (m, 1H), 5.44 (s, 2H), 4.21 (q, J = 7.2 Hz, 2H), and 1.25 (t, J = 7.1 Hz, 3H) ppm are attributed to 3f.

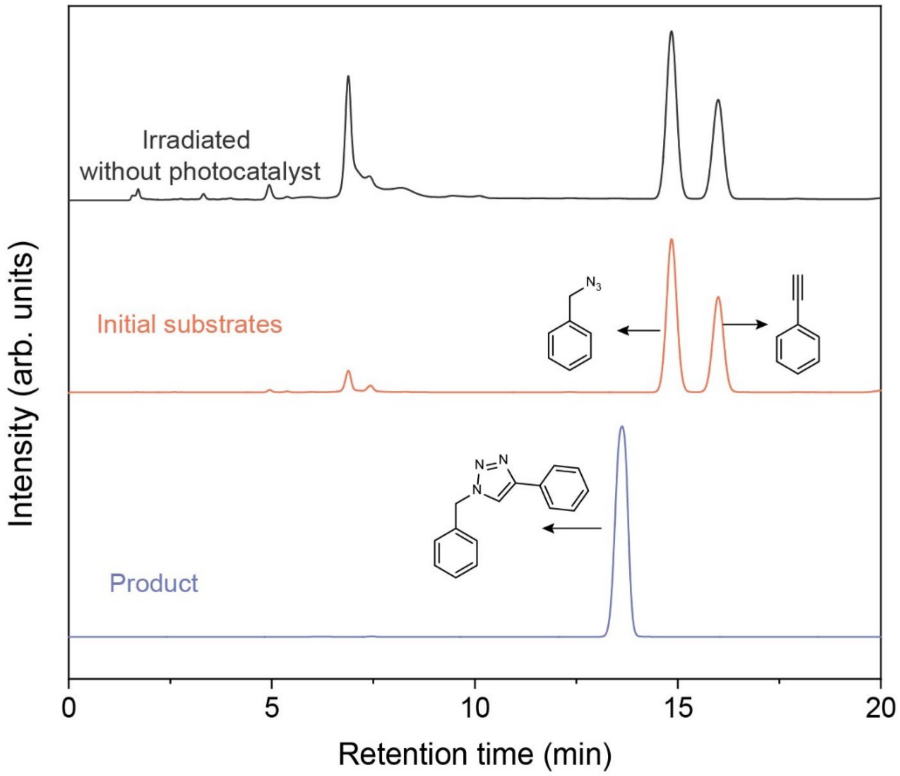

Supplementary Figure 24. The HPLC signals of pure product, substrates, and the control subjects. The retention time of benzylazide, phenylacetylene, and triazoles are centered at 14.5, 16.0, and 13.5 min, respectively. In the control experiment, the HPLC peak intensities of substrates did not change, while the peaks before a retention time of 10 min increased considerably under light irradiation without a photocatalyst.

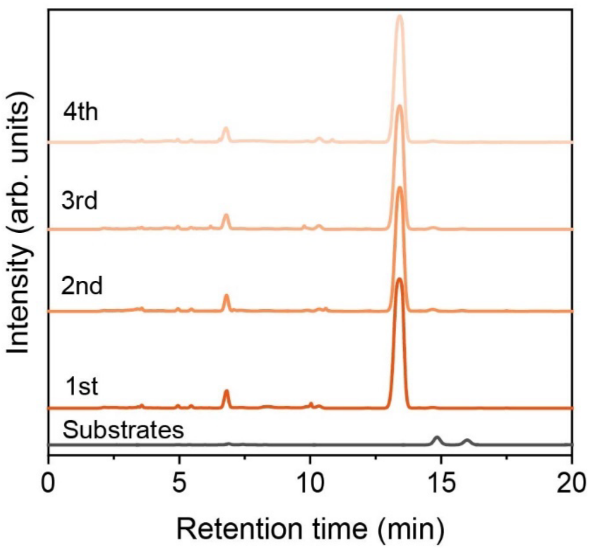

Supplementary Figure 25. The HPLC spectra of photocatalytic cycling tests. After three cycling tests, the TPB-BPy-Cu still exhibits robust photocatalytic CuAAC activity.

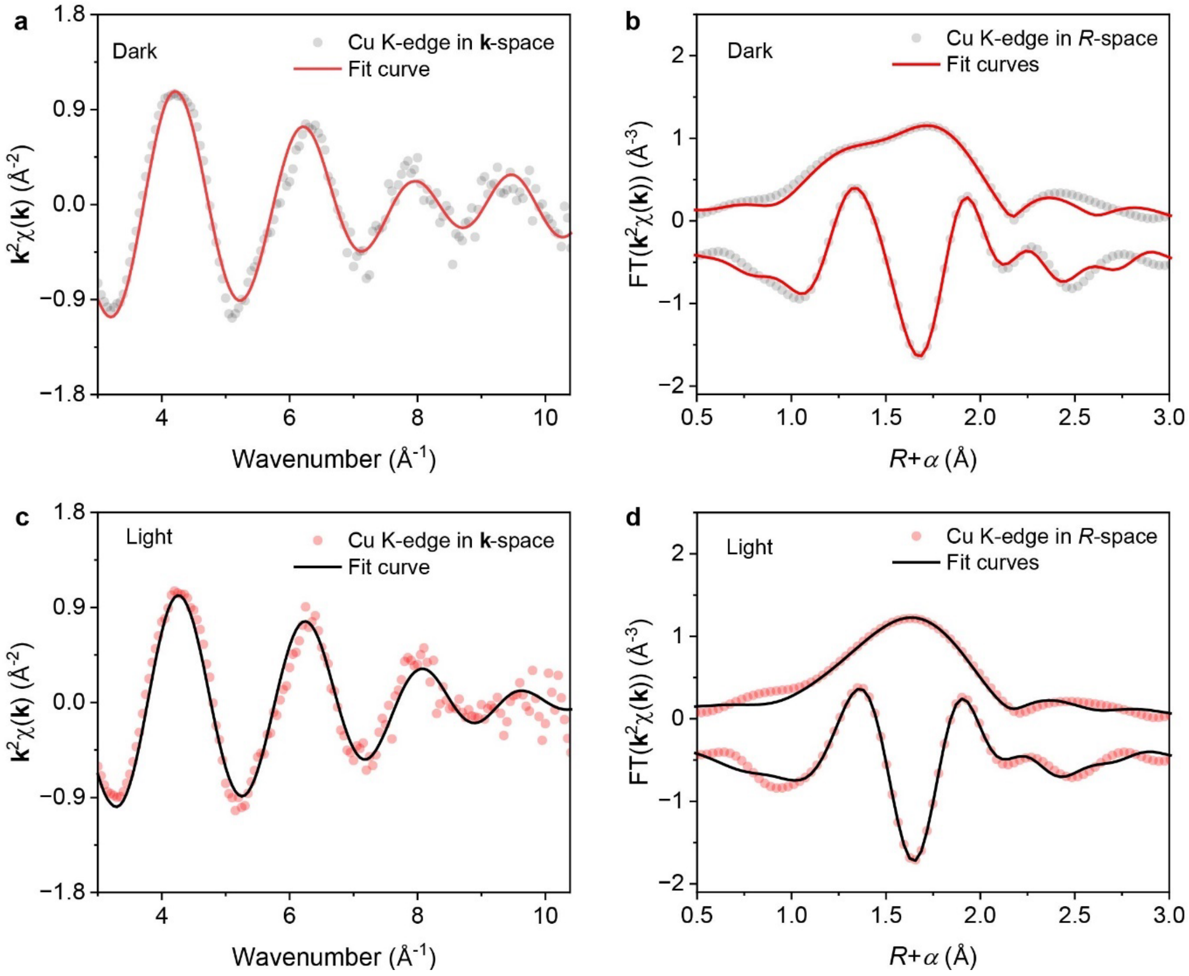

Supplementary Figure 26. In situ irradiated Cu K-edge EXAFS spectrum for TPB-BPy-Cu. The $\mathbf{k}^2$-weighted **k**-space and *R*-space (**a, b**) in dark conditions and (**c, d**) under irradiation.

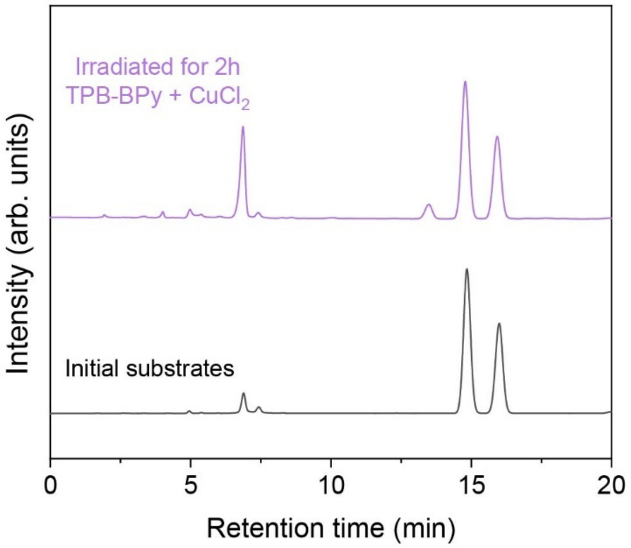

Supplementary Figure 27. The HPLC spectra of photocatalytic CuAAC over metal-free TPB-BPy in the presence of Cu(II) salt, added as $CuCl_2$ at a dosage equivalent to that in TPB-BPy-Cu.

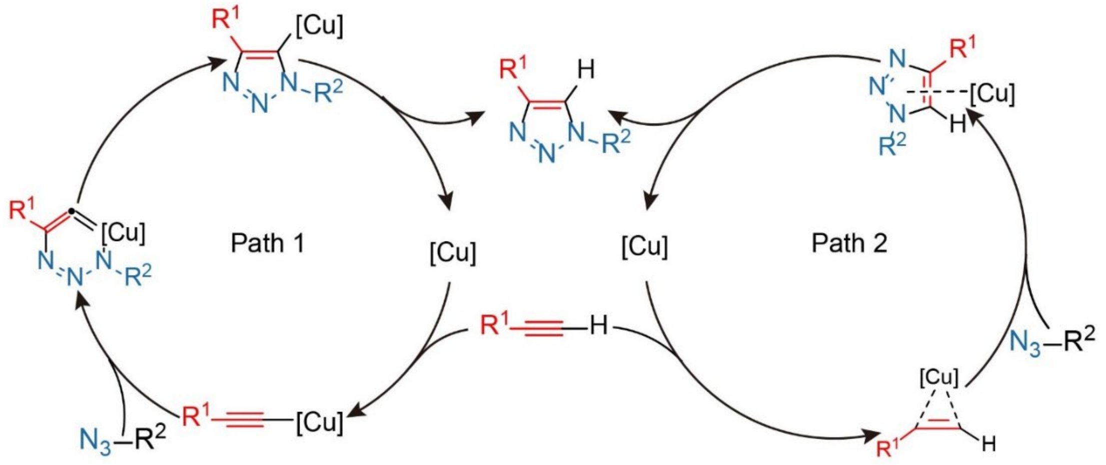

Supplementary Figure 28. Two common catalytic mechanisms for the CuAAC reaction. In the first proposed mechanism, the initial step is the formation of a Cu acetylide (C≡C-Cu). In the second mechanism, the initial step is the attack of the Cu site on the alkynyl bond instead of forming a terminal Cu-alkyne intermediate, resulting in the construction of a Cu-containing three-membered ring.

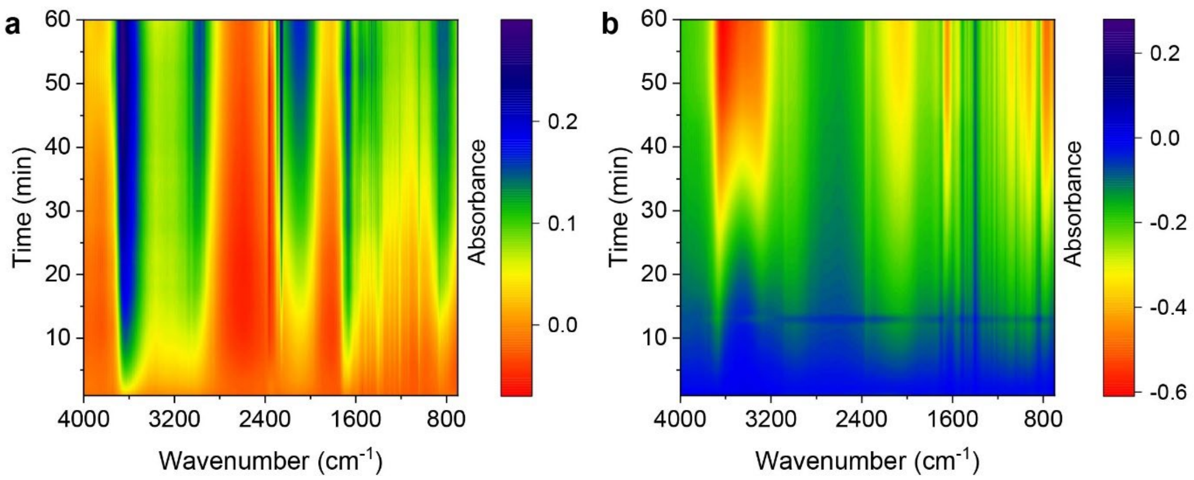

Supplementary Figure 29. Two-dimensional DRIFTS mapping of sample surface (a) in the dark and (**b**) under UV light (λ = 365 nm) irradiation. The 2D mapping spectra under dark and illumination conditions exhibit complementary color distributions, indicating that the substrates adsorbed in the dark and the intermediates are gradually consumed as light is turned on.

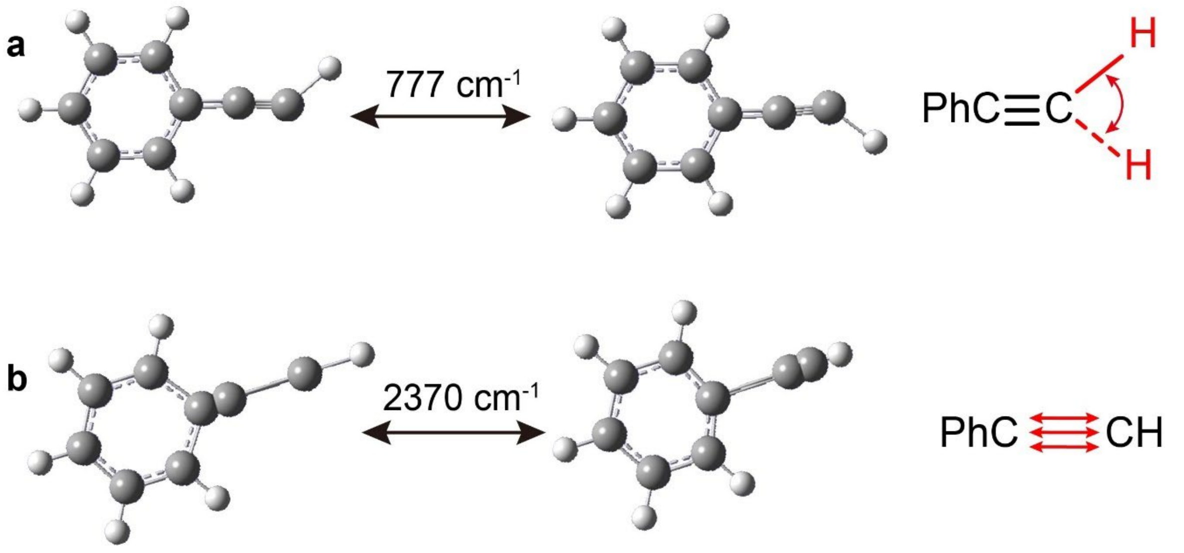

Supplementary Figure 30. Calculated IR signals of phenylacetylene. The simulated (**a**) terminal alkyne C-H bending vibration and (**b**) C≡C stretching vibration of phenylacetylene, which are located at 777 and 2370 cm^{-1}, respectively.

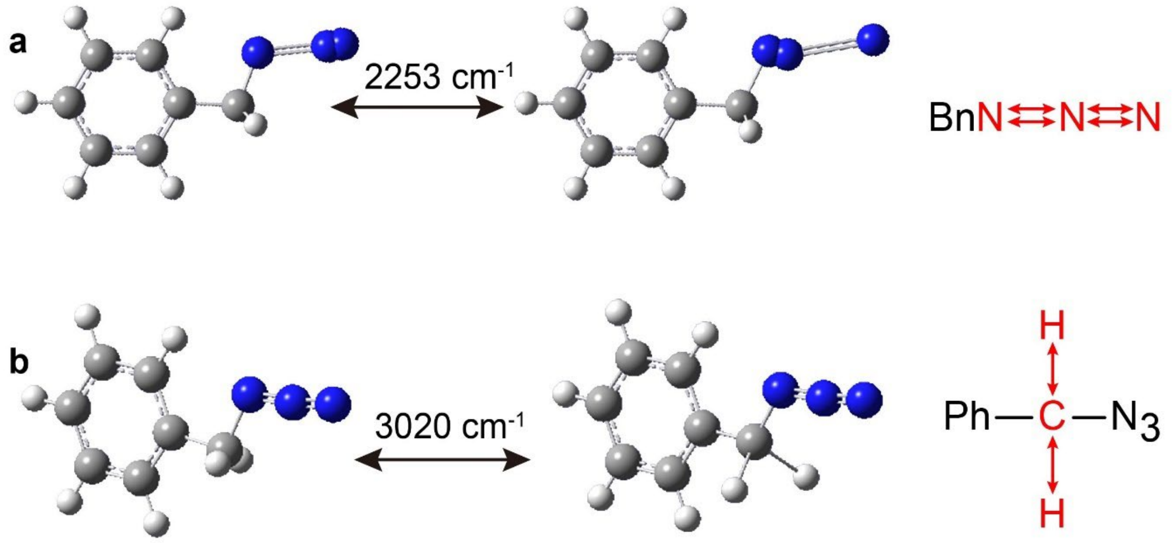

Supplementary Figure 31. Calculated IR signals of benzylazide. The simulated (**a**) N=N=N stretching vibration and (**b**) benzyl C-H stretching vibration of benzylazide, which are located at 2253 and 3020 cm^{-1} respectively.

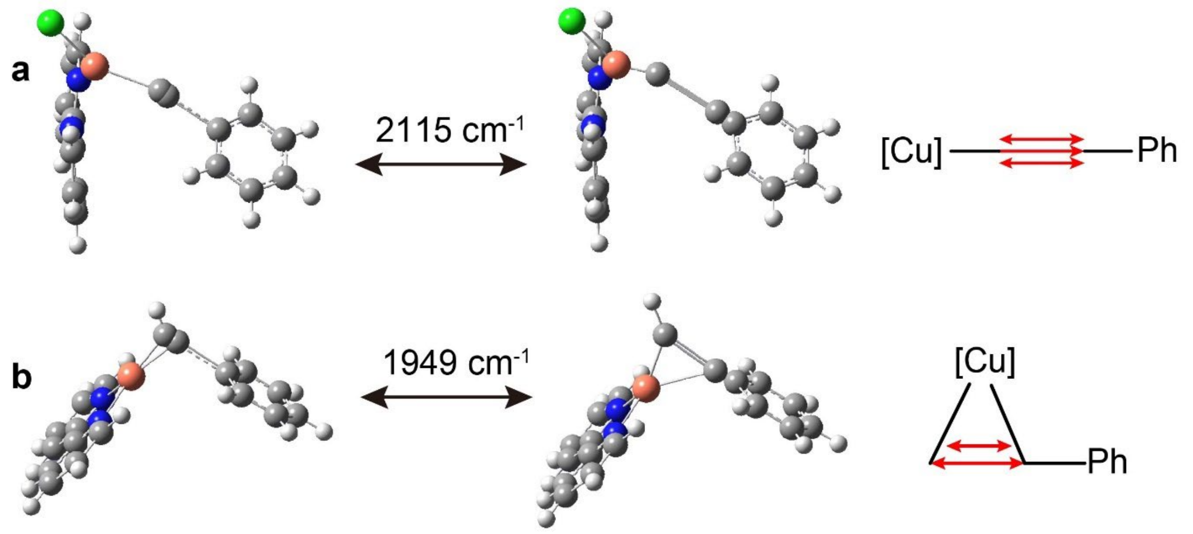

Supplementary Figure 32. The simulated vibration of potential intermediates. The characteristic vibration of (**a**) terminal C≡C-Cu and (**b**) Cu-containing three-membered ring, which are located at 2115 and 1949 cm^{-1}, respectively.

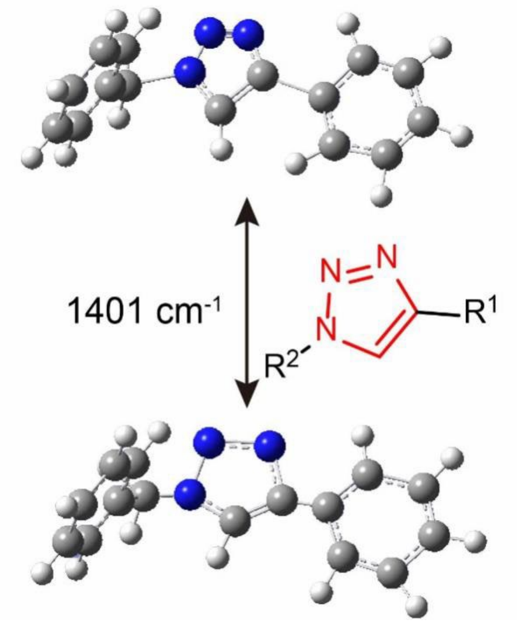

Supplementary Figure 33. Calculated IR signals of photocatalytic reaction product. The simulated in-plane bending vibration of triazole ring, located at 1401 cm^{-1}.

Supplementary Tables

Supplementary Table 1. DFT-optimized geometry of TPB-BPy-Cu fragments, computed at the level of PBE1PBE/DEF2SVP.

Atom	x	y	z	Oxidation state [a]
C(1)	–5.977	1.953	–0.835	2
C(2)	–6.000	0.543	–0.857	4
C(3)	–4.777	–0.151	–0.761	2
C(4)	–3.551	0.536	–0.638	4
C(5)	–3.564	1.946	–0.616	2
C(6)	–4.769	2.671	–0.716	4
C(7)	–4.766	4.157	–0.696	4
C(8)	–7.284	–0.195	–0.982	4
C(9)	–2.273	–0.210	–0.535	4
C(10)	–5.802	4.876	–0.058	2
C(11)	–5.799	6.277	–0.038	2
C(12)	–4.759	6.991	–0.655	2
C(13)	–3.724	6.290	–1.293	2
C(14)	–3.727	4.889	–1.313	2
C(15)	–7.493	–1.411	–0.293	2
C(16)	–8.703	–2.107	–0.410	2
C(17)	–9.733	–1.602	–1.220	2
C(18)	–9.541	–0.395	–1.911	2
C(19)	–8.331	0.301	–1.793	2
C(20)	–1.206	0.278	0.255	2
C(21)	–0.004	–0.426	0.351	2
C(22)	0.177	–1.643	–0.345	4
C(23)	–0.887	–2.138	–1.138	2
C(24)	–2.086	–1.434	–1.227	2
C(25)	1.445	–2.355	–0.242	2

N(26)	1.674	–3.470	–0.859	–3
C(27)	2.927	–4.054	–0.835	4
C(28)	3.050	–5.465	–0.842	2
C(29)	4.315	–6.050	–0.840	2
C(30)	5.460	–5.228	–0.841	4
N(31)	5.333	–3.877	–0.867	–3
C(32)	4.129	–3.300	–0.873	2
C(33)	6.852	–5.721	–0.825	4
C(34)	7.212	–7.081	–0.760	2
C(35)	8.569	–7.426	–0.760	2
C(36)	9.534	–6.409	–0.816	2
C(37)	9.105	–5.076	–0.866	2
N(38)	7.802	–4.752	–0.875	–3
H(39)	–6.924	2.506	–0.913	1
H(40)	–4.782	–1.250	–0.778	1
H(41)	–2.615	2.494	–0.523	1
H(42)	–6.607	4.326	0.453	1
H(43)	–6.610	6.817	0.474	1
H(44)	–4.757	8.091	–0.640	1
H(45)	–2.909	6.840	–1.789	1
H(46)	–2.924	4.351	–1.840	1
H(47)	–6.702	–1.801	0.366	1
H(48)	–8.847	–3.047	0.143	1
H(49)	–10.684	–2.149	–1.313	1
H(50)	–10.339	0.005	–2.556	1
H(51)	–8.181	1.232	–2.359	1
H(52)	–1.336	1.211	0.823	1
H(53)	0.815	–0.034	0.976	1
H(54)	–0.734	–3.081	–1.682	1

H(55)	–2.892	–1.818	–1.869	1
H(56)	2.215	–1.888	0.418	1
H(57)	2.136	–6.076	–0.847	1
H(58)	4.415	–7.144	–0.845	1
H(59)	4.154	–2.196	–0.959	1
H(60)	6.440	–7.860	–0.706	1
H(61)	8.869	–8.483	–0.713	1
H(62)	10.609	–6.639	–0.819	1
H(63)	9.785	–4.204	–0.882	1
Cu(64)	7.110	–2.790	–0.901	2
Cl(65)	6.090	–0.840	–1.294	–1
Cl(66)	9.126	–1.908	–0.525	–1

[a] The oxidation states were acquired through a localized orbital bonding analysis (LOBA)[1].

Supplementary Table 2. Curve fit parameters for refinement of Cu K-edge EXAFS of TPB-BPy-Cu.

Path	d (Å)	N	R (Å)	σ^2 (Å^2)
Cu-N	1.966	2	1.98	0.004
Cu-Cl	2.234	2	2.24	0.003

S_0^2 = 0.91 was fixed at the value for Cu foil measured as a reference. ΔE_0 was refined as a global fit parameter for the sample. Coordination numbers (N) were fixed at integer values to give physical meaning to the values for σ^2. Data range: $3.0 \leq \mathbf{k} \leq 10.4$ Å^{-1}, $1.0 \leq R \leq 3.0$ Å. The total number of independent data points is 9.2.

Supplementary Table 3. Calculated vertical excitation energies (Δ*E*), absorption wavelength, and oscillator strengths (*f*) of the lowest 15 excited states of TPB-BPy-Co.

Excited states	Δ*E* (eV)	Wavelength (nm)	*f*
1	0.6165	2011.10	0.0006
2	0.8224	1507.59	0.0004
3	0.9478	1308.13	0
4	1.4899	832.16	0
5	2.1439	578.31	0
6	2.6366	470.24	0.0003
7	2.6745	463.58	0.0002
8	2.7497	450.90	0.0021
**9**	**2.8414**	**436.35**	**0.0538**
10	2.8472	435.46	0.0039
**11**	**2.9483**	**420.53**	**0.7181**
12	3.0176	410.87	0.0148
13	3.0211	410.39	0.0002
14	3.0494	406.59	0.0002
15	3.1001	399.94	0.0119

The calculations were carried out using the PBE0 functional with the SVP basis set, and the bright states with the highest oscillator strengths are highlighted in bold.

There are two possible excited states (with the highest oscillator strength), which are No. 11 and 9. The energy level of excited state 11 is high-lying over excited state 9, hence they can be interpreted as the ES and LMCT state, respectively.

Supplementary Table 4. The quantified distributions of electrons and holes in excited states No. 11 and 9.

	GS→Ex 11		Ex 11→Ex 9	
	COF ligand	Cu	COF ligand	Cu
Proportion of electron distribution (%)	98.79	0.2	13.86	61.46

Similar to the charge decomposition analysis, the distributions of electrons and holes can also be divided into two parts: the distributions on the COF ligand and the Cu site. For Ex 11, distribution of photogenerated electrons are almost totally located on the COF ligand. Therefore, the transition from GS to Ex 11 represents the intrinsic excitation of COF ($\pi \rightarrow \pi^*$). As for excited state Ex 9, the distribution of photogenerated electrons are decreased on ligand but dramaticaly increased on Cu, which indicates that a considerable part of electrons in the COF ligand is injected into the Cu site.

Supplementary Table 5. Photocatalytic CuAAC performance and Cu leaching amounts in four cycling tests.

Cycles [a]	Conversion (%) [b]	Selectivity (%)	Cu leaching percentage (%) [c]
1	95	92	0.04
2	97	92	0.05
3	91	93	0.05
4	95	93	0.06

[a] Due to the low dosage of TPB-BPy-Cu (2 mg) used in the photocatalytic tests, it was difficult to fully collect the recovered catalysts. Therefore, we conducted ten sets of parallel photocatalytic reactions, increasing the total dosage of TPB-BPy-Cu to 20 mg. At the end of every cycle, we collected all solids from each batch of reactions and weighed out 2 mg of dried samples for the next cycling test. The remaining solids were divided into 9 portions and reused in subsequent parallel photocatalytic reactions.

[b] The conversion of substrates is calculated based on the concentration of benzylazide.

[c] The leaching amount of Cu was measured using ICP-OES. In detail, after each cycle reaction, the photocatalyst was removed by filtration. The collected solutions were diluted ten times using H_2O, and the concentration of Cu in the solution was determined by ICP-OES. Based on the actual content of Cu in TPB-BPy-Cu (7.15%, acquired from ICP-OES), the Cu leaching percentage can be calculated.

Supplementary Table 6. Curve fit parameters for refinement of in situ irradiated Cu K-edge EXAFS of TPB-BPy-Cu [a].

Path		*d* (Å)	*N*	*R* (Å)	σ^2 (Å^2)
Dark	Cu-N	1.966	2.00	1.88	0.001
	Cu-Cl	2.234	2.00	2.21	0.003
Light	Cu-N	1.966	2.00	1.93	0.003
	Cu-Cl	2.234	**1.97** [b]	2.13	0.005

[a] The coordination structure of Cu(II) was explored by in situ irradiated X-ray absorption spectroscopy (XAS). All the experiments were conducted under N_2 atmosphere. S_0^2 was fixed at the value (i.e., 0.91) for Cu foil measured as a reference. ΔE_0 was refined as a global fit parameter for the sample. Coordination numbers (*N*) were fixed at integer values to give physical meaning to the values for σ^2. Data range: $3.0 \leq \mathbf{k} \leq 10.4$ Å^{-1}, $1.0 \leq R \leq 3.0$ Å. The total number of independent data points is 9.2.

[b] Under the dark condition, the coordination number of TPB-BPy-Cu was fixed at 4 (2 for Cu-N and 2 for Cu-Cl), with the Cu-N bond length estimated at 1.88 Å and the Cu-Cl bond length estimated at 2.21 Å. When fitting the spectra under illumination, the coordination number of N was fixed at 2 and that of Cl was released. The estimated coordination number of Cl was 1.97, which is extremely close to the initial value (i.e., 2.00).

Supplementary References

1. Thom, A.J.W., Sundstrom, E.J., Head-Gordon, M. LOBA: a localized orbital bonding analysis to calculate oxidation states, with application to a model water oxidation catalyst. *Phys. Chem. Chem. Phys.* **11**, 11297 (2009).
