# Supplementary material for: Single-atom photocatalyst for click reaction
Source: Nat Commun. 2026 Jun 10;17:7376. doi: 10.1038/s41467-026-74278-4 (PMC13402698; doi:10.1038/s41467-026-74278-4)
Supplement: Supplementary file 2 — Peer Review file [file 41467_2026_74278_MOESM2_ESM.pdf]

nature portfolio

Peer Review File

Single-atom photocatalyst for click reaction

Corresponding Author: Professor Jiaguo Yu

This file contains all reviewer reports in order by version, followed by all author rebuttals in order by version.

Version 0:

Reviewer comments:

Reviewer #1

(Remarks to the Author)
This is an interesting piece of work by Cheng et al, where COF-Cu single atom catalysts are furuitfully used for the click reaction.
The study is conducted with a high level of experimental details, enriched with advanced techniques used for fully characterize the material. The activity reported is also excellent, so I am pretty sure that the manuscript deserves pubblication in Nat Commun upon addressing some minot issue:

1) the COFs are typically high surface materials with controlled porosity. How relevant is this for the photocatalytic reaction? Could the surface area and porosity be measured?

2) I think the loading of Cu should be measured by XPS, ICP-MS

3) Can the catalyst be recycled? Is the copper stable over repeated cycles?

Apart from these main points, I like the accuracy of the article, the quality and I find the results of good impact, so I would reccommend for acceptance

Reviewer #2

(Remarks to the Author)
Azide-alkyne click reaction is mediated by Cu(I) species. These species are sensitive to air. As such, they are generated in situ, typically using stoichiometric quantity of reductants. To avoid this issue, authors' idea is to construct COF that is functionalized with Cu single atoms, TPB-BPy-Cu. Upon excitation with light and followed by the charge separation a state of the material is generated, in which Cu atoms are in the oxidation state (I), while the photogenerated hole, presumably, rests on the ligand (COF). The Cu(I)-species immobilized within the material drive azide-alkyne click reaction. Overall, this is an interesting idea. It was explained well in the introduction and it is supported by the experimental data. Still, the catalytic part of this work should be improved for the paper to become suitable for publication in Nature Communications.
1. By using XPS and XANES, authors detected reduction of Cu(II) to Cu(I). Is this process reversible, i.e., what is the lifetime of Cu(I) in TPB-BPy-Cu? Can recovery of Cu(II) be detected by XPS and/or XANES?
2. XPS (Figure 5D) and, at some extent, XANES (Figure 5E) confirm generation of Cu(I) species triggered by irradiation with light. Authors should provide complementary spectra for C and N elements to check if 1) the photogenerated hole remains on ligand (this would give direct evidence for LMCT) OR 2) it is reduced by some species, accident sacrificial electron donors, such as chlorine ligand, for example. Concerning the second possibility, I recommend synthesizing an analogous material, in which Cl atoms at Cu are replaced by ligands, which are more stable against oxidation, such as triflate, hexafluorophosphate, etc.; and compare properties and catalytic activity of this material with that of TPB-BPy-Cu prepared from $CuCl_2$.
3. Page 17, "…suggesting that the photogenerated Cu(I) species can be easily coupled to the substrates and remain stable during the photocatalysis reaction." Can this interaction be detected by XPS and/or XANES?
4. The present study is built on the assumption that sacrificial electron is not required. Instead, illumination of TPB-BPy-Cu generates in situ Cu(I) species, which act as perpetual catalytic sites for the reaction. This implies, that continuous irradiation with light is not necessary, but only illumination of TPB-BPy-Cu for a short period of time to accumulate sufficient quantity of Cu(I) species. Can authors confirm this? If not, what is the mechanism of catalyst deactivation?
5. What are the TON and TOF values?

6. The scope of alkynes and azides should be expanded to study how the catalyst is sensitive to electronic and steric effects.

Version 1:

Reviewer comments:

Reviewer #1

(Remarks to the Author)
The authors have addressed all the issues coming from the first reviewing stage, and I think the manuscript is now ready for acceptance in Nature communications

Reviewer #2

(Remarks to the Author)
Authors conducted a thorough revision of their manuscript and addressed fully reviewers' comments. As such, I recommend publication of the manuscript in Nature Communications. It will be a nice addition to the area of research.

Open Access This Peer Review File is licensed under a Creative Commons Attribution 4.0 International License, which permits use, sharing, adaptation, distribution and reproduction in any medium or format, as long as you give appropriate credit to the original author(s) and the source, provide a link to the Creative Commons license, and indicate if changes were made.
In cases where reviewers are anonymous, credit should be given to 'Anonymous Referee' and the source.
The images or other third party material in this Peer Review File are included in the article’s Creative Commons license, unless indicated otherwise in a credit line to the material. If material is not included in the article’s Creative Commons license and your intended use is not permitted by statutory regulation or exceeds the permitted use, you will need to obtain permission directly from the copyright holder.
To view a copy of this license, visit https://creativecommons.org/licenses/by/4.0/

Reviewer #1 (Remarks to the Author):

This is an interesting piece of work by Cheng et al, where COF-Cu single atom catalysts are furuitfully used for the click reaction.
The study is conducted with a high level of experimental details, enriched with advanced techniques used for fully characterize the material. The activity reported is also excellent, so I am pretty sure that the manuscript deserves pubblication in Nat Commun upon addressing some minot issue:

1) the COFs are typically high surface materials with controlled porosity. How relevant is this for the photocatalytic reaction? Could the surface area and porosity be measured?
Response: According to the reviewer's suggestions, we have conducted the nitrogen sorption tests to measure the specific surface area and porosity of the TPB-BPy and TPB-BPy-Cu samples. As shown in Figure R1, the specific surface area of TPB-BPy was 891 $m^2 g^{-1}$, and its pore volume was 0.50 $cm^3 g^{-1}$. Upon coordination of Cu, the specific surface area and pore volume of TPB-BPy-Cu were significantly decreased to 268 $m^2 g^{-1}$ and 0.39 $m^3 g^{-1}$, respectively. These results indicate that the textural properties are not the dominant factors in affecting the photocatalytic performance of our photocatalytic system.

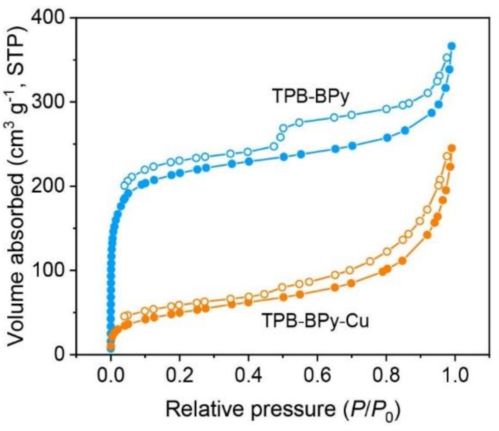

Figure R1. Nitrogen adsorption-desorption isotherms of TPB-BPy and TPB-BPy-Cu. The Brunauer–Emmett–Teller (BET) specific surface areas and pore volumes were acquired through Barrett-Joyner-Halenda (BJH) method.

2) I think the loading of Cu should be measured by XPS, ICP-MS
Response: We thank the reviewer for the constructive comment. We have measured the actual mass ratio of Cu, which was 7.15% (Supplementary Table 5), using ICP-OES (Since the Cu content was relatively high, we did not use the more sensitive ICP-MS.) We have also measured the loading of Cu by XPS, and the obtained atomic ratio of Cu was only 1.01%, which typically arises from the presence of adventitious carbon. Therefore, we turned to calculate the Cu/N atomic ratio, which can avoid the perturbation of other elements, particularly adventitious carbon. The atomic ratio of Cu/N was 1:4 as measured by XPS, which is in line with its theoretical ratio.

3) Can the catalyst be recycled? Is the copper stable over repeated cycles?

Response: As shown in Figure R2 (which is Supplementary Figure 25 in the revised manuscript), the TPB-BPy-Cu photocatalyst is recyclable and show stable photocatalytic performance. The stability of Cu can be testified by the leaching experiments. As shown in Table R1 (which is Supplementary Table 5 in the revised manuscript), only trace amount of Cu (0.2%) was leached after four photocatalytic cycles. The reviewer's concerns about stability also reminded us that we previously did not provide any experimental details of the cycling tests. We now provide the following description of the method as notes under Table R1: Due to the low dosage of TPB-BPy-Cu (2 mg) used in the photocatalytic tests, it was difficult to fully collected the recovered catalysts. Therefore, we conducted ten sets of parallel photocatalytic reactions, increasing the total dosage of TPB-BPy-Cu to 20 mg. At the end of every cycle, we collected all solids from each batch of reactions and weighed out 2 mg of dried samples for the next cycling test. The remaining solids were divided into 9 portions and reused in subsequent parallel photocatalytic reactions.

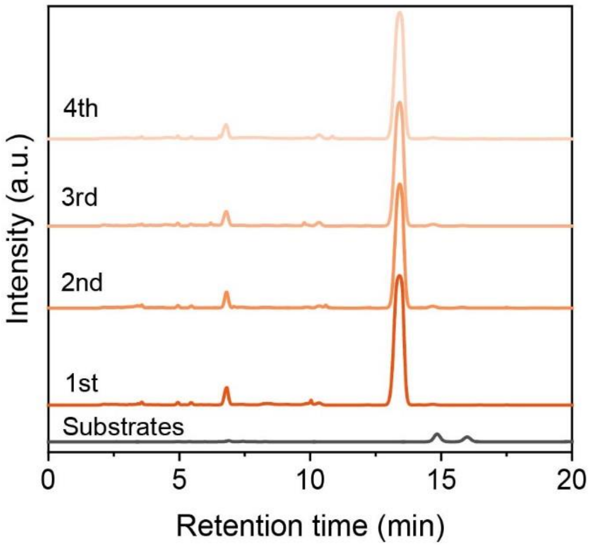

Figure R2. The HPLC spectra of photocatalytic cycling tests. After three cycling tests, the TPB-BPy-Cu still exhibits robust photocatalytic CuAAC activity.

Table R1. Photocatalytic CuAAC performance and Cu leaching amounts in four cycling tests.

Cycles [a]	Conversion (%) [b]	Selectivity (%)	Cu leaching percentage (%) [c]
1	95	92	0.04
2	97	92	0.05
3	91	93	0.05
4	95	93	0.06

[a] Due to the low dosage of TPB-BPy-Cu (2 mg) used in the photocatalytic tests, it was difficult to fully collect the recovered catalysts. Therefore, we conducted ten sets of parallel photocatalytic reactions, increasing the total dosage of TPB-BPy-Cu to 20 mg. At the end of every cycle, we collected all solids from each batch of reactions and weighed out 2 mg

of dried samples for next cycling test. The remaining solids were divided into 9 portions and reused in subsequent parallel photocatalytic reactions.

[b] The conversion of substrates is calculated based on the concentration of benzylazide.

[c] The leaching amount of Cu was measured using ICP-OES. In detail, after each cycle reaction, the photocatalyst was removed by filtration. The collected solutions were diluted ten times using H_2O, and the concentration of Cu in the solution was determined by ICP-OES. Based on the actual content of Cu in TPB-BPy-Cu (7.15%, acquired from ICP-OES), the Cu leaching percentage can be calculated.

Moreover, in response to the reviewer's inquiry, we synthesized a new batch of TPB-BPy-Cu and compared its Cu K-edge XANES before and after photocatalytic test. As shown in Figure R3A, the Cu K-edge XANES spectra before and after photocatalytic test are completely overlapped, indicating that the oxidation state of Cu remains stable. The k^3-weighted EXAFS R-space data (Figure R3B) demonstrate that the coordination structure of TPB-BPy-Cu remains unchanged after photocatalytic test.

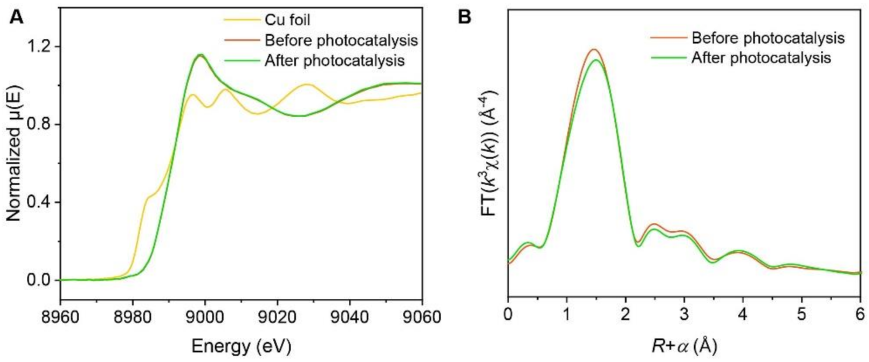

Figure R3. (A) Cu K-edge XANES spectra of TPB-BPy-Cu before and after photocatalytic test and the reference sample Cu foil. (B) Cu K-edge EXAFS spectra for TPB-BPy-Cu before and after photocatalytic test in k^3-weighted R-space.

Apart from these main points, I like the accuracy of the article, the quality and I find the results of good impact, so I would reccommend for acceptance

Reviewer #2 (Remarks to the Author):

Azide-alkyne click reaction is mediated by Cu(I) species. These species are sensitive to air. As such, they are generated in situ, typically using stoichiometric quantity of reductants. To avoid this issue, authors' idea is to construct COF that is functionalized with Cu single atoms, TPB-BPy-Cu. Upon excitation with light and followed by the charge separation a state of the material is generated, in which Cu atoms are in the oxidation state (I), while the photogenerated hole, presumably, rests on the ligand (COF). The Cu(I)-species immobilized within the material drive azide-alkyne click reaction. Overall, this is an interesting idea. It was explained well in the introduction and it is supported by the experimental data. Still, the catalytic part of this work should be improved for the paper to

become suitable for publication in Nature Communications.

1. By using XPS and XANES, authors detected reduction of Cu(II) to Cu(I). Is this process reversible, i.e., what is the lifetime of Cu(I) in TPB-BPy-Cu? Can recovery of Cu(II) be detected by XPS and/or XANES?

Response: The Cu(I) species was generated upon ligand to metal charge transfer; hence its lifetime can be interpreted as that of the LMCT state, which was 1108 ps as estimated by fs-TA spectroscopy. According to the reviewer's comment, we performed the XPS and XANES measurements under "light off-on-off" conditions. As shown in Figure R4 (which is Fig. 5D in the revised manuscript), the XPS peaks of Cu(I) 2*p* first appear upon irradiation and then disappear when the light is turned off. The in situ irradiated XANES results exhibit similar tendency (Figure R5, which is Fig. 5E in the revised manuscript). The Cu K-edge shifts toward lower energy upon irradiation and shifts back after the light source is turned off. These results indicate that the reduction of Cu(II) to Cu(I) is reversible.

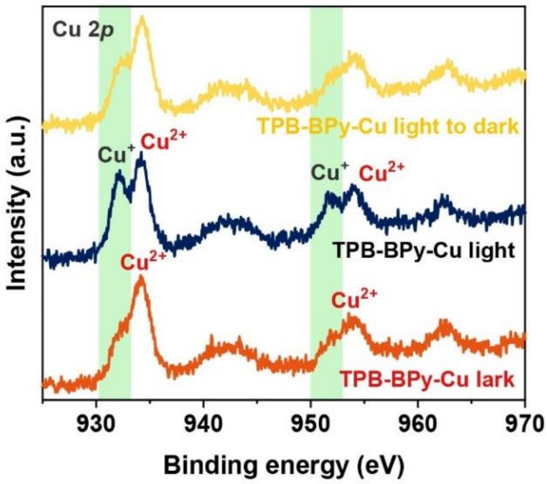

Figure R4. In situ irradiated XPS spectra of Cu 2*p* core level in TPB-BPy-Cu.

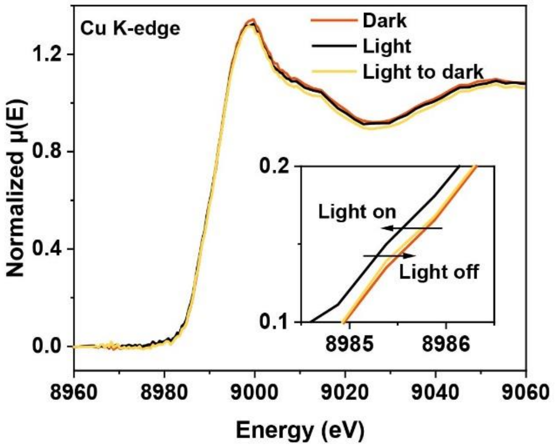

Figure R5. In situ irradiated Cu K-edge XANES spectra of TPB-BPy-Cu under dark-light-dark conditions.

2. XPS (Figure 5D) and, at some extent, XANES (Figure 5E) confirm generation of Cu(I) species triggered by irradiation with light. Authors should provide complementary spectra for C and N elements to check if 1) the photogenerated hole remains on ligand (this would give direct evidence for LMCT) OR 2) it is reduced by some species, accident sacrificial electron donors, such as chlorine ligand, for example. Concerning the second possibility, I recommend synthesizing an analogous material, in which Cl atoms at Cu are replaced by ligands, which are more stable against oxidation, such as triflate, hexafluorophosphate, etc.; and compare properties and catalytic activity of this material with that of TPB-BPy-Cu prepared from CuCl2.

Response: Following the reviewer's suggestions, we conducted in situ irradiated soft X-ray absorption spectroscopy (sXAS) measurements to monitor the electronic migration behavior of the ligands. Typically, the intensity of N K-edge XANES is proportional to the density of unoccupied N orbitals. Since the N 2*p* orbitals are hybridized with Cu 3*d* and 4*sp* orbitals, they also account for these metal orbitals. As shown in Figure R6 (which is Supplementary Fig. 13 in the revised manuscript), the N K-edge XANES of TPB-BPy-Cu can be divided into two energy regions. The region from 390 to 404 eV is assigned to N 1*s* → N(2*p*)-Cu(3*d*) transition. Theoretically, illumination should have little effect on the intensity of this transition because photoinduced LMCT occurs only between the N 2*p* and Cu 3*d* orbitals. Therefore, we normalized the peak in this region as a reference. The region from 404 to 420 eV is assigned to N 1*s* → N(2*p*)-Cu(4*sp*) transition, where the contribution of Cu 4*sp* can be neglected because the photogenerated electrons are rarely injected into this orbital; thus, the absorption intensity is directly associated with the density of unoccupied N orbitals. Upon light irradiation, the absorption intensity in the high-energy region is distinctly enhanced, suggesting that a fraction of N orbitals becomes unoccupied, which provides direct evidence for the generation of holes.

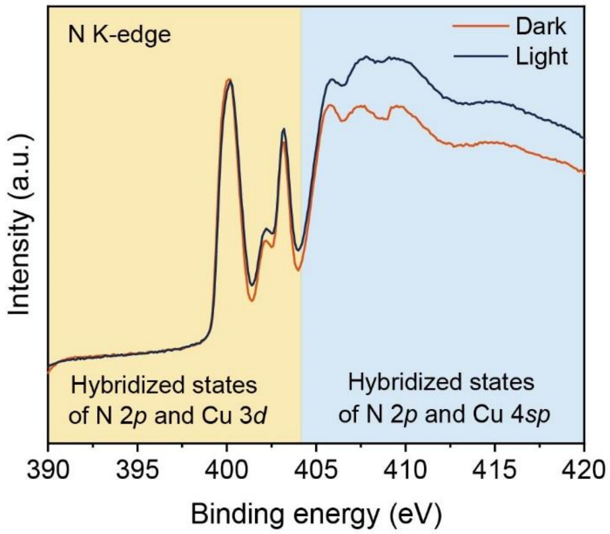

Figure R6. In situ irradiated N K-edge sXAS of TPB-BPy-Cu.

Considering the reviewer's concerns over the stability of the Cl ligand, we realized that we

had not provided a sufficient explanation for the in situ irradiated XANES data. As shown in Table R2 (which is Supplementary Table 6 in the revised manuscript), under the dark condition, the coordination number of the Cu(II) complex is fixed at 4 (2 for Cu-N and 2 for Cu-Cl), with the Cu-N bond length estimated at 1.88 Å and the Cu-Cl bond length estimated at 2.21 Å. When fitting the spectra under illumination, we fixed the coordination number of N at 2 and released that of Cl. The results showed that the estimated coordination number of Cl was 1.97, which is extremely close to the initial value (i.e., 2.00). Although the bond lengths changed, this still confirms that no Cl detachment occurs.

Table R2. Curve fit parameters for refinement of in situ irradiated Cu K-edge EXAFS of TPB-BPy-Cu [a].

Path		*d* (Å)	*N*	*R* (Å)	σ^2 (Å^2)
Dark	Cu-N	1.966	2.00	1.88	0.001
	Cu-Cl	2.234	2.00	2.21	0.003
Light	Cu-N	1.966	2.00	1.93	0.003
	Cu-Cl	2.234	**1.97** [b]	2.13	0.005

[a] The coordination structure of Cu(II) was explored by in situ irradiated X-ray absorption spectroscopy (XAS). All the experiments were conducted under N_2 atmosphere. S_0^2 was fixed at the value (i.e., 0.91) for Cu foil measured as a reference. ΔE_0 was refined as a global fit parameter for the sample. Coordination numbers (*N*) were fixed at integer values to give physical meaning to the values for σ^2. Data range: $3.0 \leq k \leq 10.4$ Å^{-1}, $1.0 \leq R \leq 3.0$ Å. The total number of independent data points is 9.2.

[b] Under dark condition, the coordination number of TPB-BPy-Cu was fixed at 4 (2 for Cu-N and 2 for Cu-Cl), with the Cu-N bond length estimated at 1.88 Å and the Cu-Cl bond length estimated at 2.21 Å. When fitting the spectra under illumination, the coordination number of N was fixed at 2 and that of Cl was released. The estimated coordination number of Cl was 1.97, which is extremely close to the initial value (i.e., 2.00).

In response to the reviewer's suggestion, we synthesized an analogous material TPB-BPy-Cu(OTf)$_2$, in which Cl atoms are replaced by triflate (OTf) groups. The experimental details are as follows:

Synthesis of BPy-Cu(OTf)$_2$. As shown in Figure R7, a propanoic acid solution (3 mL) of BPy (21.2 mg, 0.1 mmol) was dropwise added into a propanoic acid solution (3 mL) of Cu(OTf)$_2$ (36.1 mg, 0.1 mmol) under continuous stirring. A light-blue solid gradually precipitated from the solution at room temperature. After stirring for 2 h, the precipitate was collected by centrifugation and washed with hot propanoic acid (3 × 10 mL) to remove the residual Cu and bipyridine. Upon solvent removal in vacuo, the pure product was obtained (46.8 mg, 81%).

Synthesis of TPB-BPy-Cu(OTf)$_2$. The BPy-Cu(OTf)$_2$ (51.6 mg, 0.09 mmol), TPB (21.1 mg, 0.06 mmol), and acetic acid (0.2 mL) were reacted in a solvent (6 mL, mesitylene:1,4-dioxane = 2:1) to produce TPB-BPy-Cu(OTf)$_2$, with a yield of 75%.

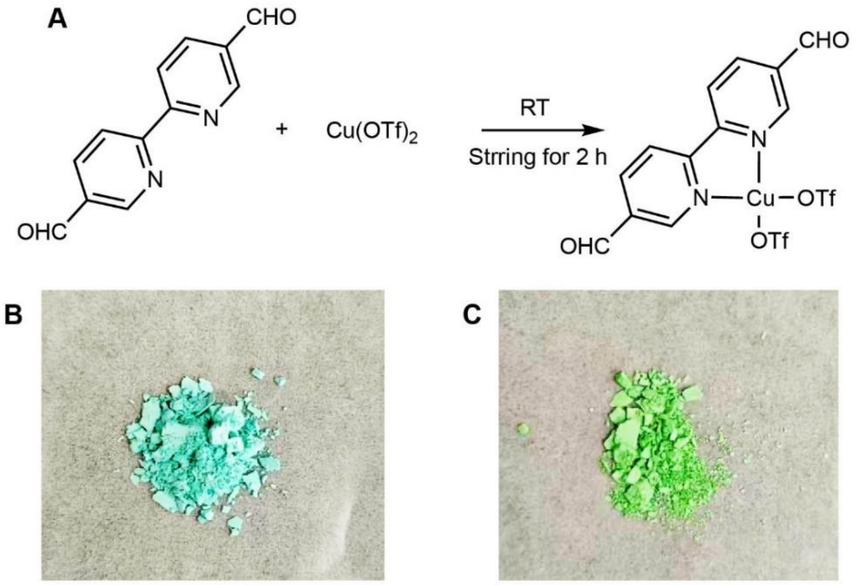

Figure R7. (A) Synthesis of BPy-Cu(OTf)$_2$. (B and C) The photographs of BPy-Cu with (B) triflate groups and (C) Cl atoms as ligands.

The photocatalytic activity of TPB-BPy-Cu(OTf)$_2$ was evaluated under the same conidtion. As shown in Figure R8, The BPy-Cu(OTf)$_2$ achieved robust substrate conversion (>95%).

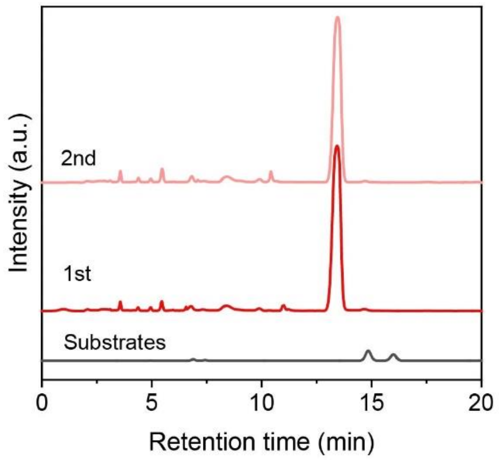

Figure R8. HPLC spectra for photocatalytic CuAAC over TPB-BPy-Cu(OTf)$_2$.

3. Page 17, "…suggesting that the photogenerated Cu(I) species can be easily coupled to the substrates and remain stable during the photocatalysis reaction." Can this interaction be detected by XPS and/or XANES?

Response: According to the reviewer's suggestion, we have carried out the in situ irradiated XANES under the atmosphere of saturated benzylazide/phenylacetylene/acetonitrile vapor. As shown in Figure R9 (which is Supplementary Fig. 16 in the revised manuscript), the Cu K-edge is further negatively shifted relative to that under N_2 atmosphere, which solidly proves the interaction between TPB-BPy-Cu and substrates.

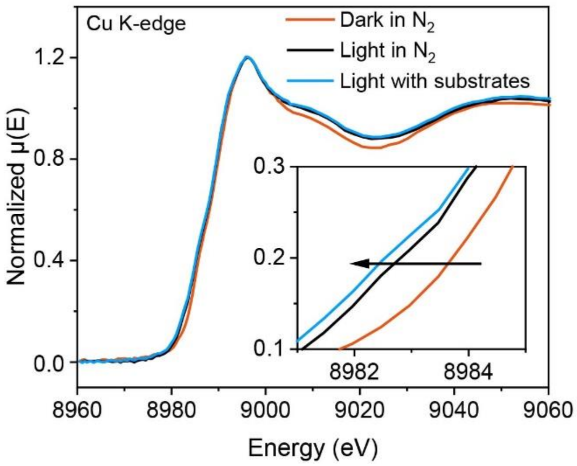

Figure R9. In situ irradiated Cu K-edge XANES spectra of TPB-BPy-Cu under N_2 atmosphere and benzylazide/phenylacetylene/acetonitrile vapor.

4. The present study is built on the assumption that sacrificial electron is not required. Instead, illumination of TPB-BPy-Cu generates in situ Cu(I) species, which act as perpetual catalytic sites for the reaction. This implies, that continuous irradiation with light is not necessary, but only illumination of TPB-BPy-Cu for a short period of time to accumulate sufficient quantity of Cu(I) species. Can authors confirm this? If not, what is the mechanism of catalyst deactivation?

Response: We would like to clarify that the in situ generated Cu(I) species cannot act as perpetual catalytic sites for the reaction, but requires continual light illumination. According to the reviewer's comment, we conducted a control experiment with the light turned off after 1-h photocatalysis test and monitored the substrate conversion before/after switching the light. As shown in Figure R10, the conversion of substrates reached 50% after 1-h irradiation. When the light was turned off, the mixture was left stirring for another hour, but the conversion of substrates was virtually unchanged, indicating that the active species for the CuAAC reaction, that is, Cu(I), was quenched. (The peak intensity of triazole was slightly increased, which could be ascribed to its desorption from the surface of TPB-BPy-Cu.) These results solidly proved that the catalytic reaction requires continuous illumination.

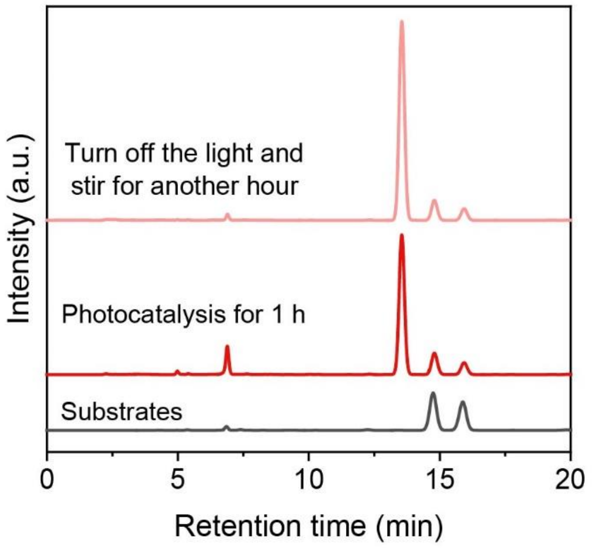

Figure R10. The HPLC spectra of photocatalytic CuAAC over TPB-BPy-Cu before/after switching the light.

The instability of Cu(I) species under dark conditions can be understood as follows. Under illumination, π bonding electrons overcome the energy barrier between π and π* orbitals, and subsequently be injected into the high-energy Cu(II) 3*d* orbital located above the π-bonding orbital. In this photoexcited state, the total energy of the system is significantly higher than that of TPB-BPy-Cu in its ground state. In the absence of light, this unstable system can spontaneously decay to the ground state, with the electrons transferring from Cu(I) 3*d* orbital back to the ligand. From a steady-state perspective, this deactivation mechanism is in line well with the results of in situ irradiated XPS and XANES tests (Figures R4 and R5). From a microscopic perspective, the spontaneous decay manifests as the transient lifetime (1108 ps) of the LMCT state.

5. What are the TON and TOF values?

Response: We have calculated the turnover number (TON) and turnover frequency (TOF) values for TPB-BPy-Cu.

Typically, the TON is calculated by equation R1:

$$\mathrm{TON} = \frac{\mathrm{Number\ \ of\ \ converted\ \ molecules}}{\mathrm{Number\ \ of\ \ active\ \ sites}} \quad \text{(R1)}$$

Notably, in homogeneous catalysis systems, the number of active sites typically equals the number of molecular photocatalysts. However, it hard to calculated the number of active sites in our reaction system with polymeric photocatalysts. Benefiting from the well-defined Cu single atoms, we estimate the number of active sites from the total amount of Cu atoms in the reaction system. This leads to a conservative estimation (i.e., underestimation) of the TON (and TOF) values, since only the fraction of Cu atoms that are photoreduced to Cu(I) serve as the active site for the CuAAC reaction. According to ICP-OES test results, the actual percentage of Cu in TPB-BPy-Cu is 7.15%. Hence, there are 0.143 mg of Cu

atoms in 2 mg of samples, namely, the number of active sites is 2.25 μmol. In a photocatalytic cycle, the number of converted molecules is 95 μmol, therefore the TON can be calculated as 42.2.

The TOF is calculated by equation R2:

$$\mathrm{TOF} = \frac{\mathrm{TON}}{\mathrm{Reaction\ \ time}} \qquad \text{(R2)}$$

Accordingly, the TOF can be calculated as 21.1 h^{-1} in one photocatalytic cycle.

6. The scope of alkynes and azides should be expanded to study how the catalyst is sensitive to electronic and steric effects.

Response: To explore whether TPB-BPy-Cu is sensitive to electronic and steric effects, we expanded the substrates to 3-ethynylthiophene, 2-ethynylpyridine and a chain-like ethyl 2-azidoacetate. As displayed in Figures R11 to R16 (which are Supplementary Figs. 17 and 19-23 in the revised manuscript), alkynes containing electron-withdrawing (pyridine) and electron-donating (thiophene) groups also exhibited high reactivity. On the other hand, both aromatic and chain-like azides achieved excellent yields for the corresponding triazoles.

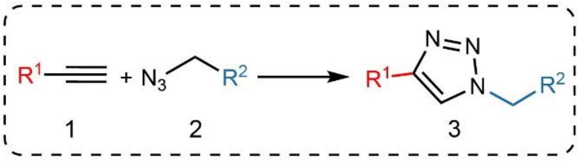

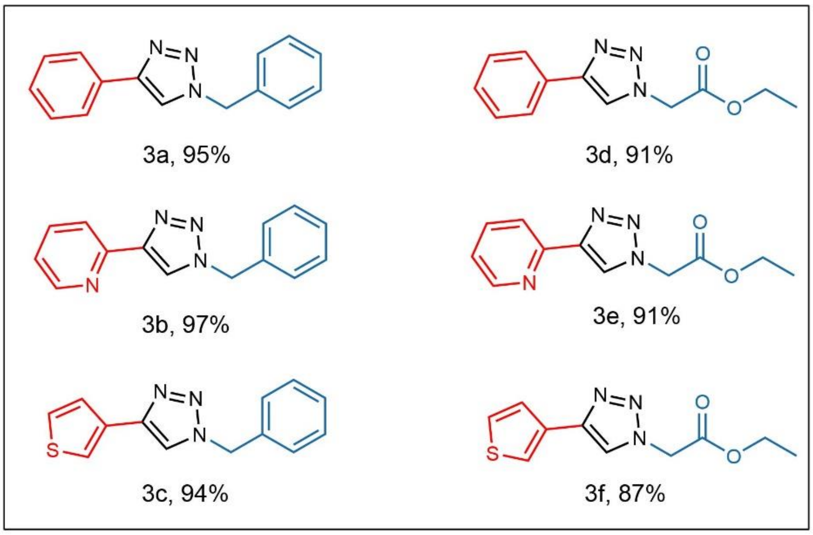

Figure R11. Schematics for photocatalytic CuAAC reaction with different substrates and the yields of different products (3a to 3f) under the same condition.

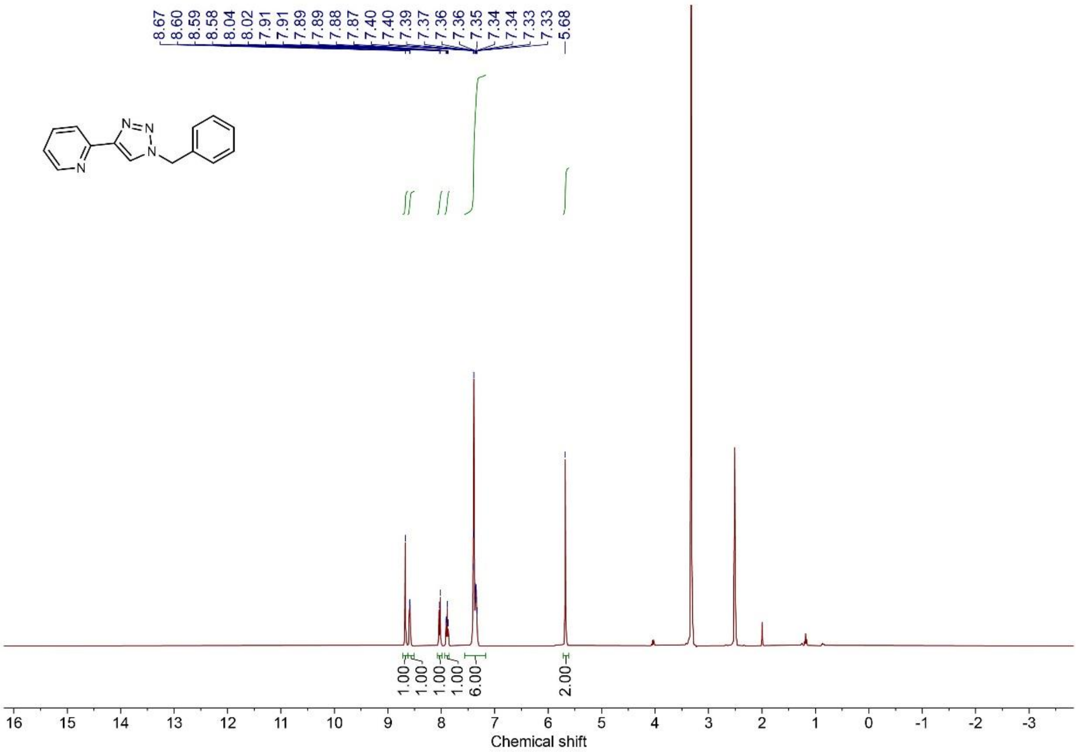

Figure R12. The ^{1}H NMR (400 MHz, DMSO) chemical shift at 8.67, 8.60, 8.59, 8.58, 8.04, 8.02, 7.91, 7.91, 7.89, 7.89, 7.88, 7.87, 7.40, 7.40, 7.39, 7.37, 7.36, 7.36, 7.35, 7.34, 7.34, 7.33, 7.33, and 5.68 ppm are attributed to 3b.

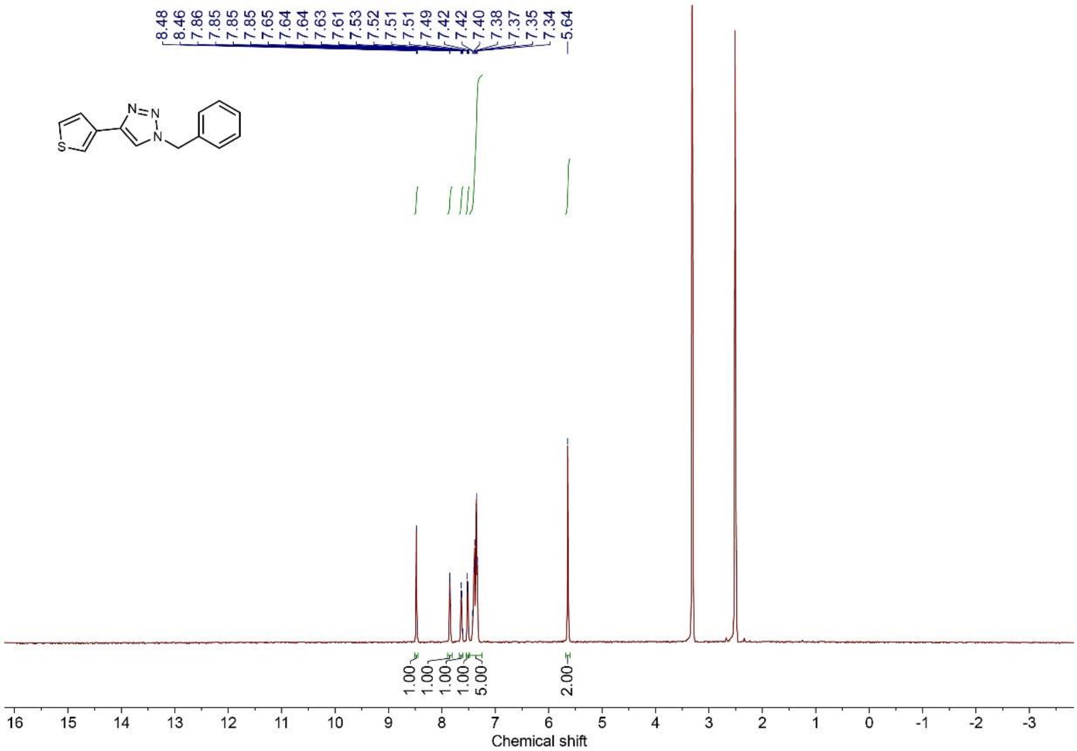

Figure R13. The ^{1}H NMR (400 MHz, DMSO) chemical shift at 8.48, 8.46, 7.86, 7.85, 7.85,

7.85, 7.65, 7.64, 7.64, 7.63, 7.61, 7.53, 7.52, 7.51, 7.51, 7.49, 7.42, 7.42, 7.40, 7.38, 7.37, 7.35, 7.34, and 5.64 ppm are attributed to 3c.

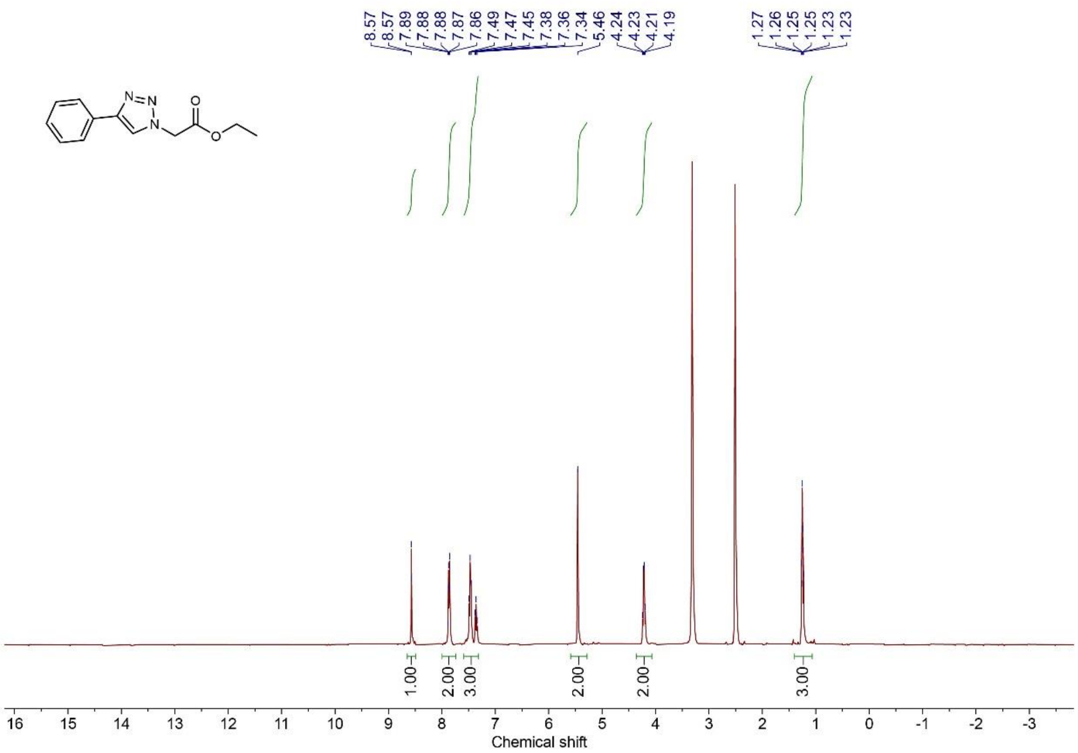

Figure R14. The ^{1}H NMR (400 MHz, DMSO) chemical shift at 8.57, 8.57, 7.89, 7.88, 7.88, 7.87, 7.86, 7.49, 7.47, 7.45, 7.38, 7.36, 7.34, 5.46, 4.24, 4.23, 4.21, 4.19, 1.27, 1.26, 1.25, 1.25, 1.23, 1.23. ppm are attributed to 3d.

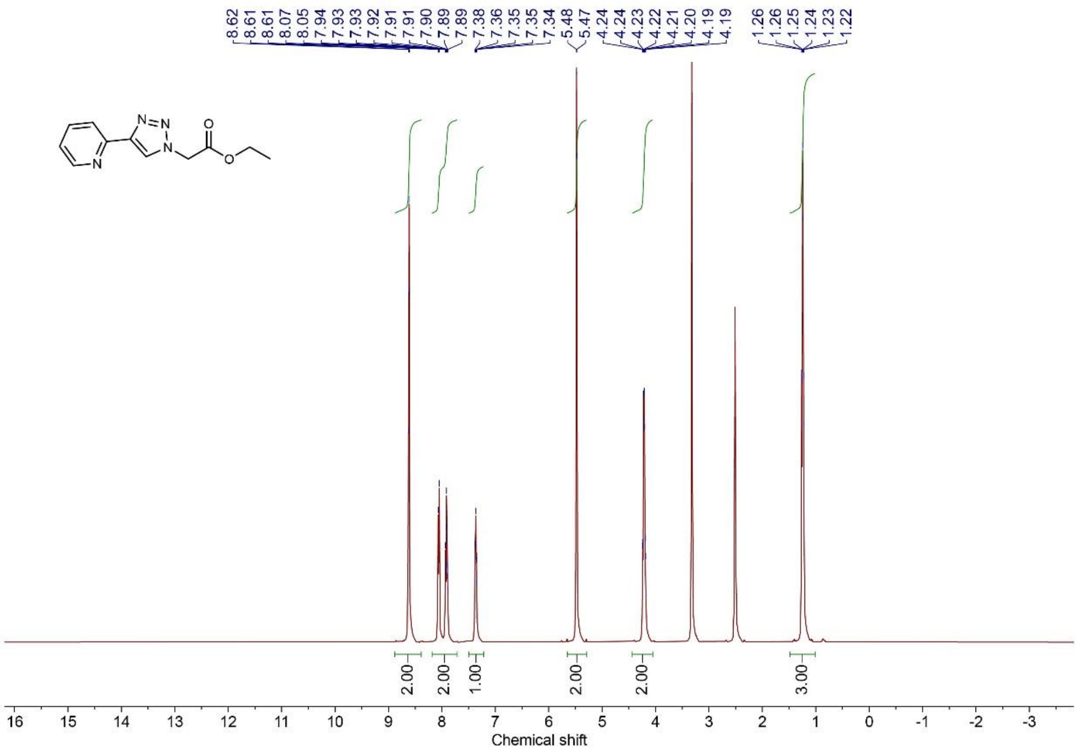

Figure R15. The ^{1}H NMR (400 MHz, DMSO) chemical shift at 8.62, 8.61, 8.61, 8.07, 8.05, 7.94, 7.93, 7.93, 7.92, 7.91, 7.91, 7.90, 7.89, 7.89, 7.38, 7.36, 7.35, 7.35, 7.34, 5.48, 5.47, 4.24, 4.24, 4.23, 4.22, 4.21, 4.20, 4.19, 4.19, 1.26, 1.26, 1.25, 1.24, 1.23, and 1.22 ppm are attributed to 3e.

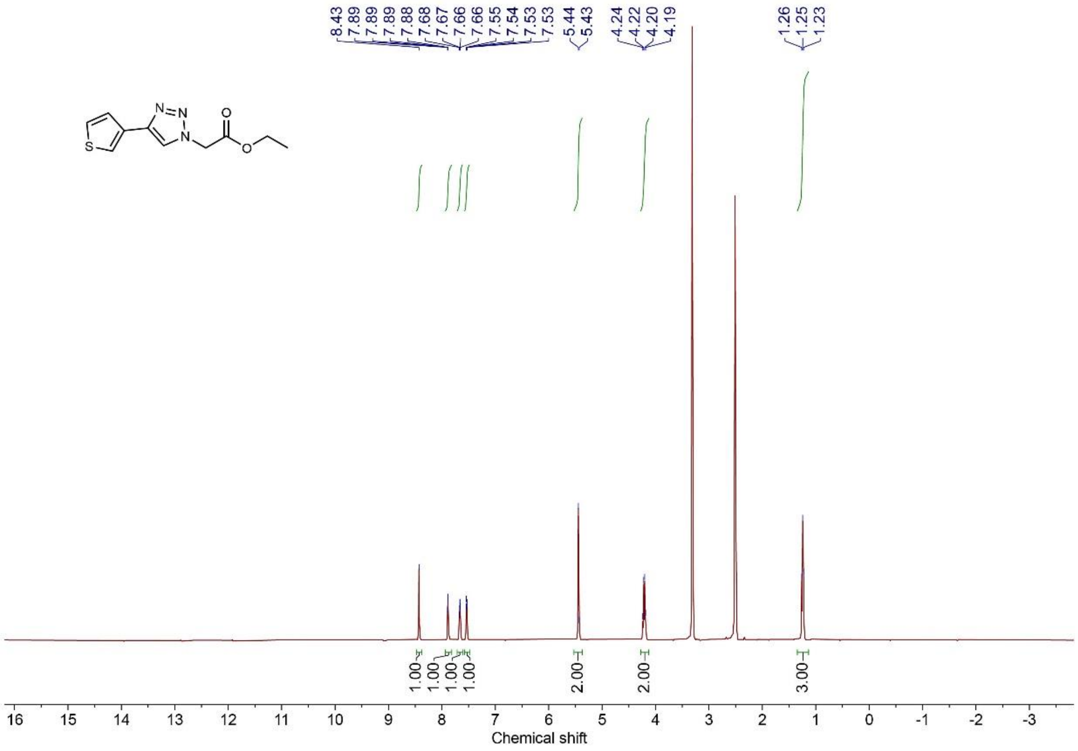

Figure R16. The ^{1}H NMR (400 MHz, DMSO) chemical shift at 8.43, 7.89, 7.89, 7.89, 7.88,

7.68, 7.67, 7.66, 7.66, 7.55, 7.54, 7.53, 7.53, 5.44, 5.43, 4.24, 4.22, 4.20, 4.19, 1.26, 1.25, and 1.23 ppm are attributed to 3f.
